# TCF7L2 acts as a molecular switch in midbrain to control mammal vocalization through its DNA binding domain but not transcription activation domain

**DOI:** 10.1038/s41380-023-01993-5

**Published:** 2023-02-13

**Authors:** Huihui Qi, Li Luo, Caijing Lu, Runze Chen, Xianyao Zhou, Xiaohui Zhang, Yichang Jia

**Affiliations:** 1 Tsinghua-Peking Joint Center for Life Sciences, Tsinghua University, Beijing, 100084 China; 2 School of Medicine, Tsinghua University, Beijing, 100084 China; 3 IDG/McGovern Institute for Brain Research, Tsinghua University, Beijing, 100084 China; 4 Tsinghua Laboratory of Brain and Intelligence (THBI), Tsinghua University, Beijing, 100084 China; 5 School of Life Sciences, Tsinghua University, Beijing, 100084 China; 6 Key Laboratory of Birth Defects and Related Diseases of Women and Children of Ministry of Education, Sichuan University, Chengdu, China; 7 State Key Laboratory of Cognitive Neuroscience and Learning, IDG/McGovern Institute for Brain Science, Beijing Normal University, Beijing, 100875 China

**Keywords:** Neuroscience, Autism spectrum disorders

## Abstract

Vocalization is an essential medium for social signaling in birds and mammals. Periaqueductal gray (PAG) a conserved midbrain structure is believed to be responsible for innate vocalizations, but its molecular regulation remains largely unknown. Here, through a mouse forward genetic screening we identified one of the key Wnt/β-catenin effectors TCF7L2/TCF4 controls ultrasonic vocalization (USV) production and syllable complexity during maternal deprivation and sexual encounter. Early developmental expression of TCF7L2 in PAG excitatory neurons is necessary for the complex trait, while TCF7L2 loss reduces neuronal gene expressions and synaptic transmission in PAG. TCF7L2-mediated vocal control is independent of its β-catenin-binding domain but dependent of its DNA binding ability. Patient mutations associated with developmental disorders, including autism spectrum disorders, disrupt the transcriptional repression effect of TCF7L2, while mice carrying those mutations display severe USV impairments. Therefore, we conclude that TCF7L2 orchestrates gene expression in midbrain to control vocal production through its DNA binding but not transcription activation domain.

## Introduction

Vocalization plays a fundamental role in intraspecific communication in songbirds, rodents, non-human primates and human [1]. In songbirds, some learning of vocal patterns, including songbird courtship call, are believed to rely heavily on imitation. In contrast, in rodent, vocalizations evoked by sexual cues or emotional states are considered to be largely innate. For example, mouse pups emit ultrasonic vocalizations (USVs) when separated from their mom, and adult male mice produces USVs in the presence of females. Such innate vocalization networks are located in the caudal brainstem and gated by the periaqueductal gray (PAG) region in the midbrain [1–3]. In rodent and non-human primates PAG has been widely considered as a key region of vocal production [1, 4]. Recently, silencing of PAG-residing neurons that are transiently active during courtship vocalization revealed that these are both necessary and sufficient for USV production [3]. Although human uses motor cortex to control the initiation and execution of speaking, bilateral lesion of the PAG in human also impairs speech and innate vocalization [1, 5, 6]. Therefore, deciphering the neural and molecular basis of PAG underlying mammal vocalization has potential implications for human language disorders.

Human speech disorders have been linked with dysfunction of relatively few transcriptional factors (TFs) and their downstream genes [7–11]. Point mutations of *FOXP2*, the most well-known vocalization gene, causes familiar language disorders and affected individuals exhibit deficits in production of words and complexity of syntax [7]. Interestingly, misregulation of the *FOXP2* gene also impair mouse USV production [12–15], underscoring common neuronal and molecular basis underlying human and mouse vocalization. To ensure it, we generated *Foxp2* KO allele (Δ5) by Crispr/Cas9 approach and measured the pup USVs after maternal deprivation with a commercial USV detector (Fig. S1). In agreement with the previous studies, *Foxp2* KO dosage-dependently impaired the USV productions at the age points we examined. In contrast, the wildtype C57BL/6 J littermates emitted reliable USVs especially at P5 and P7.

In contrast to FOXP2, genetic alternations in Transcription Factor 7 Like 2 (*TCF7L2)*, the focus on the current study, is reportedly associated with human diseases, including metabolic disorders and cancers [16, 17]. In addition, previous genome-wide analysis revealed that rare *TCF7L2* mutations are associated with developmental disorders, like autism spectrum disorders (ASDs) [18–22], and that *TCF7L2* common intronic polymorphisms are associated with neuropsychiatric disorders, like schizophrenia [23–25] and bipolar disorder [26]. Previous studies also demonstrated that loss of TCF7L2 disrupts neuronal circuitry in a way that may explain the link between nicotine addiction and diabetes, and may play a role in the etiology of neuropsychiatric disorders [27–29]. TCF7L2 belongs to a family of T-cell factor/lymphoid enhancer (TCF/LEF) transcription factors (TCF7, LEF1, TCF7L1, and TCF7L2) that are key mediators of Wnt/β-catenin signaling in numerous cellular processes ranging from early development to adult tissue homeostasis [30–33]. TCF7L2 contains a β-catenin-binding domain (CBD) for β-catenin-mediated transcriptional activation and a high-mobility group (HMG) domain for DNA binding. In the absence of nuclear β-catenin, a TCF7L2-containing transcriptional repression complex is assembled to inhibit downstream gene expression [32, 34]. Like other TCF/LEF family members, *TCF7L2* produces both full-length (flTCF7L2) and dominant-negative TCF7L2 (dnTCF7L2), the latter of which contains HMG box but not the CBD domain and therefore inhibits Wnt/β-catenin signaling cascade in a dominant negative manner [32, 33, 35, 36].

Although the neuroanatomical basis for vocalization is well studied in rodents, its molecular basis is largely unknown. Here, by using a N-ethyl-N-nitrosourea (ENU)-induced mutagenesis screening, we identified *Tcf7l2* acts as a gatekeeper for mouse USV production and syllable complexity. We show that *Tcf7l2* is highly expressed in midbrain VGLUT2 (vesicular glutamate transporter 2)-positive neurons and that its expression is necessary and sufficiently for mouse USV production; midbrain region-specific knockout (KO) of *Tcf7l2* impairs mouse USV production and *Tcf7l2* KO in VGLUT2-positive neurons decreases synaptic transmission in PAG. Mice carrying patient mutations associated with developmental disorders display significant impairments of USV production but not autism-like behaviors. In addition, like the ENU-induced Y337H, patient non-synonymous mutations associated with developmental disorders relieve the transcriptional repression effect of TCF7L2. Therefore, we conclude that TCF7L2 functions as a molecular switch in the midbrain that controls vocalization production and syllable complexity, probably through a transcriptional repression mechanism.

## Results

### An ENU-induced mutagenesis screening for genes involved in mouse vocalization

**Fig. 1:**
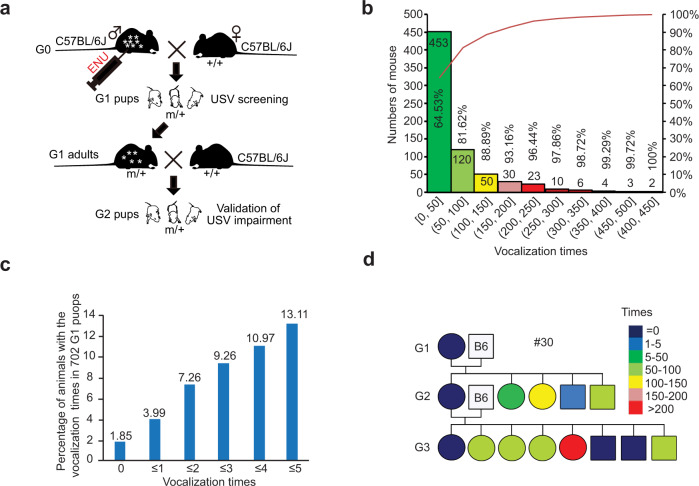
Identification of novel genes involved in mouse USV by an ENU-induced mutagenesis screen. **a** A G1 dominant screening was carried out for identification of novel genes involved in mouse USVs. G0 males were treated with ENU and then crossed to C57BL/6 J wildtype (+/+) females. Spontaneous USVs of G1 pups induced by maternal deprivation were measured by a commercial USV detector (Med Associates Inc.). We screened for pups with USV impairments. The grownups of G1 pups with USV impairment were bred to C57BL/6 J wildtype (+/+) mice. The reoccurrence of USV impairment in G2 and G3 pups was employed to establish the family pedigree. **b**, **c** The spontaneous USV number distribution of G1 pups (702) measured by the detector (Med Associates Inc.) in a 5-minute interval at P5. The G1 pups with USV number ranged from 0–5 are illustrated (**c**). **d** An ENU family pedigree (#30 family) with an inheritable USV impairment at P5.

To identify novel genes involved in mouse vocalization, we set up an ENU-induced mutagenesis screening (Fig. 1a). To this end, we crossed ENU-treated G0 males with untreated C57BL/6 J females, and through the use of an USV detector, identified G1 pups with a few or no USVs (<5 times in a 5-minute interval at P5). Out of 702 G1 pups, 610 (86.9%) emitted USVs over 5 times whereas 92 (13.1%) emitted USVs 5 times or less, of which 13 mice (1.85%) were mute (Fig. 1b, c). To establish family pedigrees of the ENU-induced mutations, we then crossed the adult USV-impaired G1 mice with untreated C57BL/6 J mice to assess phenotypic reoccurrence in G2 and G3 pups. Among several families carrying inheritable USV impairments, we consistently found mute pups in the family #30 (Figs. 1d and S1).

### ENU-induced Y337H mutation abolishes HMG box DNA binding ability

**Fig. 2:**
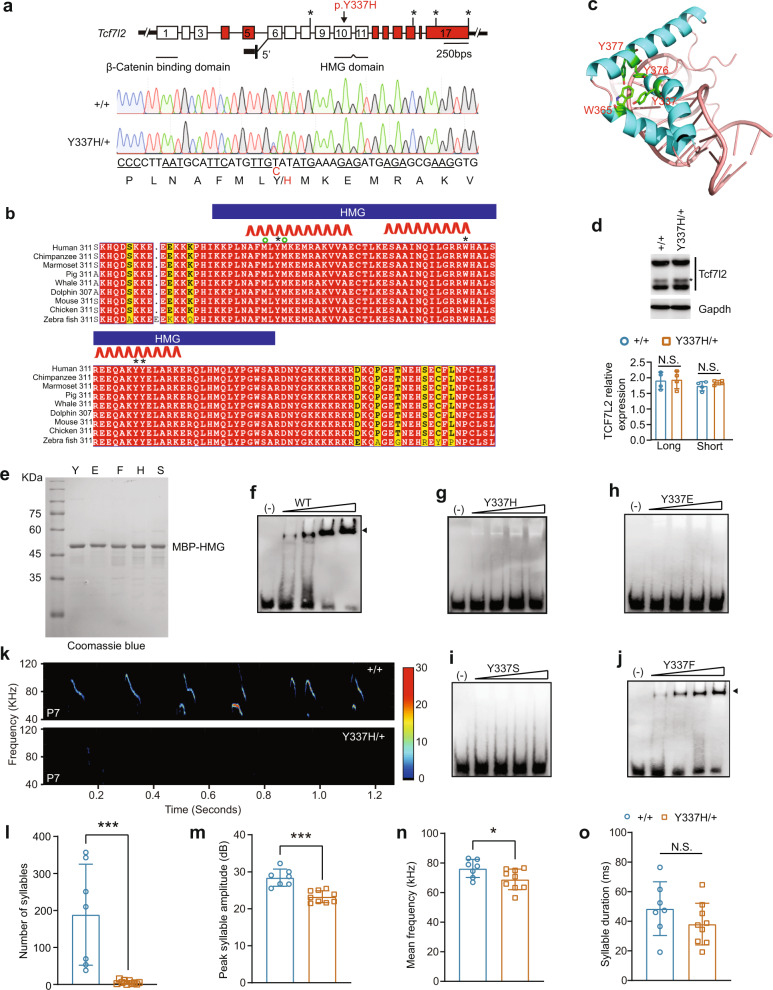
Y337H abolishes TCF7L2 DNA binding ability and impairs pup USVs. **a** The genome structure of mouse *Tcf7l2*. The alternatively spliced exons were marked in red. The ENU-induced nonsynonymous mutation identified in family #30 (c.T1019C, p.Y337H, ENSMUST00000111656.7) is located in *Tcf7l2* exon 10 that encodes part of HMG box. DNA chromatogram illustrates the mutation (Y337H/ + ) (lower). **b** The sequence conservation of TCF7L2 HMG box and its neighboring residues. *, Y337, W365, Y376, and Y377; ^O^, M335 and M338; red ribbon, alpha helix segments. **c** The Y337 and neighboring residues were adapted into previously reported HMG box/DNA interface. The hydrophobic core formed by Y337, W365, Y376, and Y377 is highlighted. **d** The expression of TCF7L2 in wildtype (+/+) and mutant (Y337H) midbrain from 4-month old mice. GAPDH, as a loading control. TCF7L2 appeared two major bands (Short and Long) in thalamus (upper) and statistic analysis of the relative expression level of these two bands (lower) in + */+* and *Tcf7l2*^Y337H/+^ mice. The anti-TCF7L2 antibody used here was generated by a synthetic peptide corresponding to sequences in HMG box. *, the major smaller band (Short). **e**–**j** HMG/DNA binding ability measured by EMSA. WT and the various mutant TCF7L2 HMG proteins fused with MBP-tag were purified by anti-MBP beads (**e**). **k** Representative spectrogram of USVs produced by + */+* and *Tcf7l2*^Y337H/+^ pups detected by MUPET. **l**–**o** The key features of USVs produced by + */+* and *Tcf7l2*^Y337H/+^ pups at P7. The value are presented as mean ± SD. In **d**, *n* = 4 (+/+ and Y337H/ + ); in **l**–**o**, +/+, *n* = 7, Y337H/ + , *n* = 8; *t*-test, SPSS. N.S., no significant difference.

To identify the mutation causing the impaired USVs in the family #30, we conducted whole-exome sequencing of DNA from affected pups. Among the examined candidate mutations, we found that only a T to C non-synonymous mutation in the *Tcf7l2* gene (c.T1019C, p.Y337H, ENSMUST00000111656.7) is always co-segregated with the USV impairment (Figs. 2a and S2). Notably, the T to C mutation changes the conserved 337 tyrosine, that has a hydrophobic side chain, into a positively charged histidine in the HMG box domain. The 337 tyrosine residue is located between two methionine residues that are proposed to be essential for HMG-mediated binding to the minor grove and bending the DNA double helix [37] (Fig. 2b). Specifically, the four aromatic residues Y12, W40, Y51, and Y52 in the HMG domain of LEF1 in murine form a hydrophobic core that stabilizes the HMG structure [37]. As illustrated in Fig. 2b, c, the Y12, W40, Y51, and Y52 residues correspond to Y337, W365, Y376, and Y377 in TCF7L2. Furthermore, the corresponding residues are also present in two other TCF/LEF family members (Fig. S3), underscoring the functional importance of these hydrophobic residues. Importantly, the ENU-induced Y337H mutation does not change TCF7L2 expression levels in mouse brain (Fig. 2d).

W40 and Y51 mutations are known to impair LEF1 HMG DNA binding [37]; however, whether the Y12 mutation impacts on the DNA binding ability of the HMG domain of TCF/LEF family members is unknown. To answer this question, we mutated Y337 to: i) glutamic acid (e) to introduce a negative charge, ii) phenylalanine (f) to introduce a hydrophobic side chain), iii) histidine (h) to introduce a positive charge, and iv) serine (S) to introduce hydrophilic side chains (Fig. 2e). In electrophoresis mobility shift assays (EMSA), we confirmed dose-dependent DNA binding of WT HMG (Fig. 2f). However, we found that the HMG/DNA binding ability was abolished in Y337H as well as Y337E mutants (Fig. 2g, h), suggesting that an impairment of HMG/DNA binding by Y337H is not due to a change in charge property. The HMG/DNA binding ability was also abolished in the Y337S mutant but largely retained by Y337F mutant HMG (Fig. 2i, j), suggesting that the hydrophobic property of Y337 is crucial for the HMG/DNA binding. Taken together, our data suggest that the ENU-induced Y337H mutation disrupts TCF7L2 binding to its target DNA, primarily by impairing formation of the HMG hydrophobic core domain.

### Y337H impairs pup vocalization and adult male vocal performances in a female-associated context

Animals heterozygous for the ENU-induced mutation (Y337H/ + ) are viable and fertile with normal brain morphologies at P7 and 4 months of age (Fig. S4a, b). As assessed by open field and Rotarod tests, *Tcf7l2*^*Y337H*/+^ mice also exhibit normal motor abilities relative to littermate wildtype controls (+/+) (Fig. S4c–e). However, severe USV abnormalities are evident in mutant pups (Fig. 2k–o), including significantly fewer syllable numbers, lower peak amplitude, and reduced mean frequency, as detected by MUPET, an open-access USV analyzer [38].

**Fig. 3:**
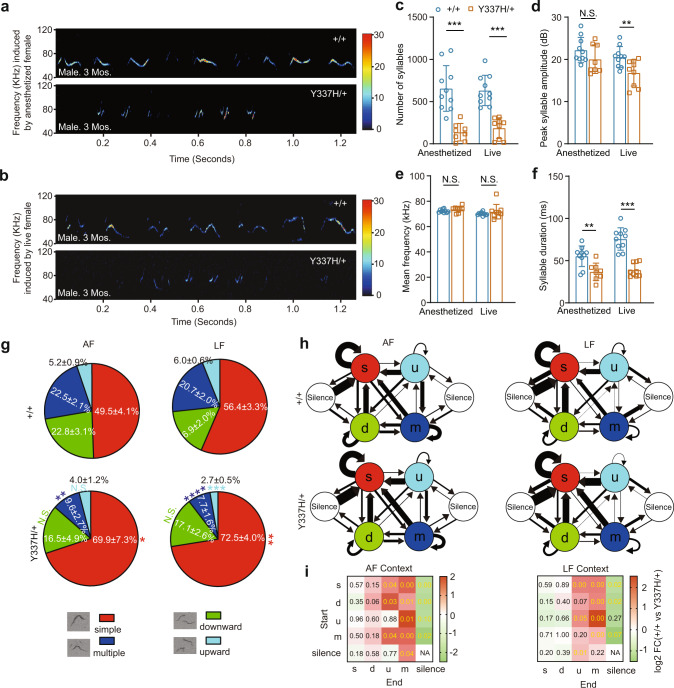
Y337H impairs adult male vocal performances in a female-associated context. Representative spectrogram of USVs emitted by the virgin + */+* or *Tcf7l2*^Y337H/+^ male in the contexts of anesthetized (AF, **a**) or live/awake (LF, **b**) virgin female (WT). Mouse age, 3 months. **c**–**f** The key features of USVs shown in (**a**) and (**b**). Different repertoire compositions of syllables (**g**) and different conditional syllable transition probabilities (**h**) between + */+* and *Tcf7l2*^Y337H/+^ males with anesthetized (AF) or live/awake WT females (LF) shown in (**a**) and (**b**). In **h**, arrow direction and thickness represent the sequence and probability of syllable transition, respectively. Four syllable categories as previously described (PMID: 25883559): red, simple; green, downward; light blue, multiple; dark blue, upward. In g, the representative spectrograms of the four type syllables were inserted. **i** Statistic analysis of conditional syllable transition probability between + */+* and *Tcf7l2*^Y337H/+^ virgin males. Number represents *p* value; color represents log2 value of the foldchange (FC). The value are presented as mean ± SD. In **c**–**f**, **g**, and **h**, +/+, *n* = 10, *Tcf7l2*^*Y337H/+*,^ *n* = 8; *t*-test, SPSS. N.S., no significant difference.

Distinct from pup vocalization, adult male and female mice emit USVs when they meet each other and the syllable complexity of such USVs is known to impact on the quality of male and female mouse social interactions [39, 40]. Here, we reliably recorded male USVs when we put + /+ virgin males together with WT virgin females, irrespective of whether the females were anesthetized (AF) - and therefore unable to produce USVs upon meeting male mice - or live/awake (LF) (Fig. 3a–f). However, when we put virgin *Tcf7l2*^*Y337H/+*^ males with awake females, we detected significantly fewer syllable numbers, decreased peak amplitude, and shorter duration (Fig. 3a–f), indicative of vocal communication defects in a female-associated context. Relative to +/+ virgin males, the *Tcf7l2*^*Y337H/+*^ males also produced significantly fewer syllable numbers of shorter duration in the presence of anesthetized females. Taken together, we conclude that the Y337H mutation impairs generation of USVs in adult males upon meeting female mice.

Next, we analyzed the syllables emitted by the *Tcf7l2*^*Y337H/+*^ or + /+ males in the presence of either awake or anesthetized females (Fig. 3g). As previously described [39], we categorized the syllables into 4 major types, including simple (s), multiple (m), downward (d), and upward (u). Relative to the + /+ virgin males, the *Tcf7l2*^*Y337H/+*^ males generated significantly more ‘s’ but fewer ‘m’ syllables in both settings (Fig. 3g). In addition, the *Tcf7l2*^*Y337H/+*^ males sang significantly fewer ‘u’ syllables in the presence of awake but not anesthetized females (Fig. 3g). Taken together, these data support that the Y337H mutation lowers the capability of generating syllable complexity in a female-associated context.

When we investigated the probability of mouse syllable transition, which reflects mouse ‘syntax’ to some degree [39], we observed that fewer syllable transitions ended up with ‘m’ type in *Tcf7l2*^*Y337H/+*^ virgin males (Fig. 3h). Moreover, more transitions ended up with ‘silence’ type in the mutant males, indicative of an early stop in the ‘syntax’ sequence (Fig. 3h, i). To quantify syllable transitions, we calculated transition probability from one type of syllable to all types with a fixed starting syllable. This approach avoids bias caused by extremely low numbers of syllable types, frequently seen in the *Tcf7l2*^*Y337H/+*^ animals [39]. In the presence of anesthetized females, we found that both syllable transition probabilities starting with any types and ending up with ‘m’ and starting with ‘s’, ‘d’, and ‘m’ types and ending up with ‘u’ were significantly higher in + /+ than in *Tcf7l2*^*Y337H/+*^ virgin males (Fig. 3h, i). Similarly, in the presence of awake females, syllable transition probabilities from ‘s’, ‘d’, ‘u’, and ‘m’ to ‘m’ and from ‘s’, ‘u’, and ‘silence’ to ‘up’ category were significantly higher in + /+ than in *Tcf7l2*^*Y337H/+*^ virgin males (Fig. 3h, i). In contrast, in both contexts, the transition probabilities from ‘s’, ‘d’, and ‘m’ to ‘silence’ were significantly lower in + /+ than in *Tcf7l2*^*Y337H/+*^ males. Based on these data, we conclude that the Y337H mutation impairs ability to complete specific syllable transitions, which breaks ‘syntax’ sequence continuity and causes premature stop.

### Haploinsufficiency of *Tcf7l2* impairs mouse vocalization

The Y337H mutation does not affect TCF7L2 expression (Fig. 2d) but abolishes DNA binding ability (Fig. 2g), suggesting that TCF7L2 carrying the Y337H mutation impairs mouse vocalization through a loss-of-function mechanism. Next, we generated a *Tcf7l2* KO mouse model by using Crispr/Cas9 technology to introduce a two-nucleotide deletion into *Tcf7l2* exon 10 (Fig. S5a, b). Heterozygous *Tcf7l2* KO mice (*Tcf7l2*^+/-^) are viable and fertile with reduced expression of high and low molecular weight TCF7L2 in the midbrain (Fig. S5c). Similar to the ENU-induced Y337H mutation (Fig. 2k–o), *Tcf7l2* haploinsufficiency severely impaired USV generation (including significantly less syllable numbers, peak amplitude, and mean frequency) relative to control animals (Fig. S5d). Unlike + /+ virgin males produces robust USVs encountering + /+ virgin females, *Tcf7l2*^*+/-*^ virgin males displayed significantly fewer syllable numbers, decreased peak syllable amplitude, and lower mean frequency with awake + /+ females (Fig. S5e).

Because the Y337H mutation in the ENU model lowers male syllable complexity and breaks their ‘syntax’ sequence continuity (Fig. 3), we asked whether this vocalization defect would affect male/female social interaction. To answer this question, we modified a three-chamber test by restricting virgin male mice to two separate sides and allowing virgin female to freely access the two males and show their preference, as previously described [3]. In this setting, we found that the female mice spent significantly more sniffing time with the wildtype males relative to both *Tcf7l2*^*Y337H/+*^ and *Tcf7l2*^*+/-*^ male mice (Fig. S6a–d). In addition, based on the preference index, females also preferred spending time with wildtype over both *Tcf7l2*^*Y337H/+*^ and *Tcf7l2*^*+/-*^ male mice (Fig. S6b, d). Our RNA-seq analysis revealed a similar RNA profiling between *Tcf7l2*^*Y337H/+*^ and *Tcf7l2*^*+/-*^ in thalamus (r = 0.89) and midbrain (r = 0.83) (Fig. S7a, b). Taken together, these studies demonstrate that like Y337H mutation, *Tcf7l2* haploinsufficiency affects pup USV production and adult vocal communication, and that Y337H mutation leads to the vocal phenotypes through a loss-of-function mechanism.

### Expression of TCF7L2 in *Vglu2*-positive neurons is sufficient and necessary for mouse vocalization

**Fig. 4:**
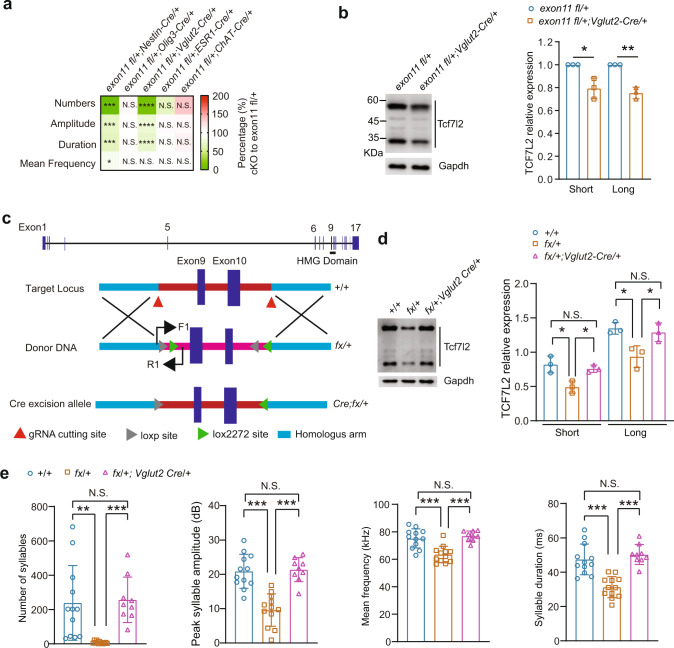
Expression of *Tcf7l2* in *Vglut2*-positive neurons is necessary and sufficient for mouse USV production. **a** The key features of pup USVs produced by the indicated *Tcf7l2* cKO pups at P7. *Nestin-*, *Oligo3-*, *Vglut2*-, *Esr1*-, and *ChAT*-*Cre* were employed to conditionally remove *Tcf7l2*. **b** The expression level of TCF7L2 in the midbrains of *exon11 fl*/+;*Vglut2*-*Cre*/+ mice by western blot at P7. The *exon11 fl*/+ mice served as controls; GAPDH, loading control. **c** A *Cre*-dependent FLEx switch allele (fx) containing an inverted *Tcf7l2* exon 9 and 10, which encode part of HMG domain, was flanked with inward-facing tandem Lox sites (LoxP:Lox2272). **d** Expression level of TCF7L2 in midbrain in the indicated genotypes at P7. In the absence of Cre recombinase, the expression level of TCF7L2 was significantly reduced in the *fx*/+ mice, which was restored by crossing to *Vglut2*-*Cre*. **e** Cre expression in *Vglut2*-positive neurons restores the pup USVs shown in the *fx*/+;*Vglut2*-*Cre*/+ mice at P7. In **a**, **b**, **d**, and **e**, data are presented as mean ± SD. N.S., no significant difference, *p < 0.05, **p < 0.01, ***p < 0.001, *t*-test or ANOVA, SPSS. In a, *Tcf7l2 exon11 fl*/+ mice were employed as controls (*n* = 7–15); *Tcf7l2 exon11 fl*/+;*Nestin*-*Cre*/+ (*n* = 12); *Tcf7l2 exon11 fl*/+;*Oligo3*-*Cre*/+ (*n* = 8); *Tcf7l2 exon11 fl*/+;*Vglut2*-*Cre*/+ (*n* = 13); *Tcf7l2 exon11 fl*/+; *Esr1*-*Cre*/+ (*n* = 7); *Tcf7l2 exon11 fl*/+;*ChAT*-*Cre*/+ (*n* = 9). In b and d, n = 3. In e, +/+ (*n* = 12); *Tcf7l2 fx*/+ (*n* = 12); *Tcf7l2 fx*/+;*Vglut2*-*Cre*/+ (*n* = 9).

Through immunostaining and in situ hybridization analysis, we demonstrate that TCF7L2 expression is concentrated in mouse thalamus and midbrain at P7 (Fig. S8a, b) consistent with a previous report [41], and that *Tcf7l2*-expressing cells are *Vglut2*-positive excitatory neurons (Fig. S8b, c). Quantitative-PCR and immunoblot analysis further confirmed that *Tcf7l2* mRNA and TCF7L2 protein expression are concentrated in thalamus and midbrain at P7 (Fig. S8d, e). In all age points examined, we detected two molecular weight TCF7L2 (Short, ~35kD; Long, ~60kD), both of which were significantly reduced in *Tcf7l2* heterozygous KO ( + /-) midbrain (Fig. S5c). Next, we employed a previously reported *Tcf7l2* flox allele, in which the loxP sites flank exon 11 that partly encodes for the HMG box [42], to determine which *Tcf7l2*-expressing cell type is responsible for mouse vocalization. Similarly to what we observed in the *Tcf7l2*^*+/-*^ mouse, midbrain expression of both short and long forms of TCF7L2 were reduced in *exon11 fl*/+;*Nestin-Cre*/+ mice (Fig. S9a). Consequently, USVs in *exon11 fl*/+;*Nestin-Cre*/+ P7 pups displayed significantly fewer syllables, reduced peak amplitude, lower mean frequency, and shorter duration than controls (Fig. 4a), indicating that *Tcf7l2* expression in neuronal progenitor is required for pup USVs.

Because *Tcf7l2* mRNA and TCF7L2 protein are highly expressed in the thalamus, we next asked whether thalamic expression of *Tcf7l2* is responsible for mouse vocalization. To this end, we generated *exon11 fl*/+;*Olig3-Cre*/+ mice, in which the cKO of *Tcf7l2* is achieved by the thalamic *Olig3-Cre* driver [43]. Although expression of both short and long TCF7L2 were reduced in the thalamus of *exon11 fl*/+;*Olig3-Cre*/+ mice (Fig. S9b) and the thalamic *Olig3-Cre* expression were confirmed (Fig. S9c), USVs were normal (Fig. 4a), indicating that expression of *Tcf7l2* in the *Olig3*-positive neurons is not a requirement for pup USVs.

Recent work revealed that estrogen receptor 1 (ESR1)-expressing neurons in the preoptic area (POA) and the ventromedial hypothalamus (VMH) participate in mouse USV [44–47]. In addition, motor neuron pools in the brain stem and spinal cord that control laryngeal and respiratory muscles also contribute to vocalization in mammals [2, 48, 49]. Here, we found that pups derived from *exon11 fl*/+ mice crossed with *Esr1*-*Cre*/+ or *ChAT*-*Cre*/+ mice emitted normal syllable numbers, peak amplitude, mean frequency, and duration at P7 (Fig. 4a). These experiments support that TCF7L2 expression in *Esr1*- or *ChAT*-positive neurons is not required for USV generation.

Given that *Tcf7l2*-expressing neurons are *Vglut2*-positive, we next generated *exon11 fl*/+;*Vglut2*-*Cre*/+ mice and validated that levels of both short and long TCF7L2 were reduced in midbrain in these mice (Fig. 4b). Relative to P7 *exon11 fl*/+ control mice, we detected significantly fewer syllable numbers, lower peak amplitude, and shorter duration in *exon11 fl*/+;*Vglut2*-*Cre*/+ pups (Fig. 4a), indicating that expression of *Tcf7l2* in *Vglut2*-positive neurons is necessary for pup vocalization. To examine whether *Tcf7l2* expression in *Vglut2*-positive neurons is also sufficient for mouse vocalization, we generated a *fx* allele for *Tcf7l2* (Fig. 4c), which inverts exon 9 and 10 and places the inward-facing Lox2272:LoxP sites flanking the two inverted exons (Fig. 4c). With this approach, we could switch on *Tcf7l2* expression in a Cre-dependent manner [50]. In the absence of *Cre*, we validated that the expression of both short and long TCF7L2 was significantly reduced in the midbrain of the *fx*/+ mice; however, the expression of TCF7L2 was restored in the midbrain of *fx*/+;*Vglut2*-*Cre*/+ mice (Fig. 4d). Importantly, the pup USV abnormalities present in the *fx*/+ mice were absent in *fx*/+;*Vglut2*-*Cre*/+ mice (Fig. 4e). Therefore, expression of TCF7L2 in *Vglut2*-positive neurons is both necessary and sufficient for mouse vocalization

### Midbrain expression of *Tcf7l2* is required for mouse vocalization

**Fig. 5:**
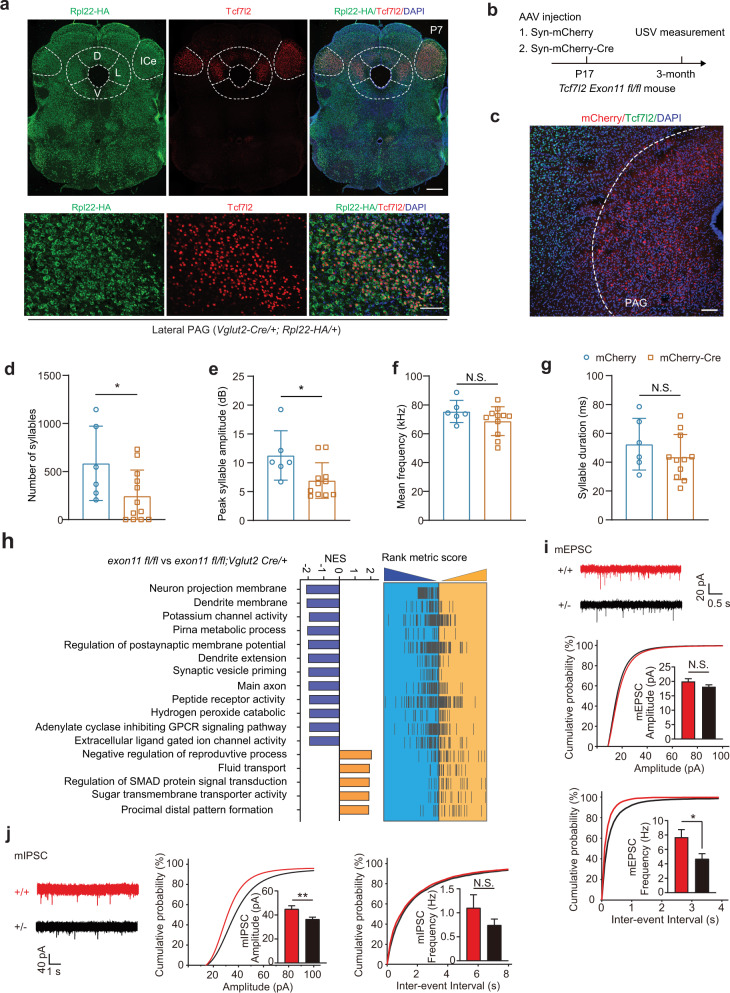
Expression of *Tcf7l2* in midbrain is required for mouse USV production and TCF7L2 loss leads to impairment of synaptic transmission in PAG. **a** The RiboTag mice (PMID: 19666516) were crossed to *Vglut2-Cre*/+ mice to label *Vglut2-*positive neurons in midbrain PAG. Neurons with TCF7L2 immunoreactive nuclear signals were positive for RPL22-HA cytosolic signals in PAG at P7 (bottom). Scale bar, 500 μm (top) and 50 μm (bottom). L, lateral; D, dorsal; V, ventral; ICe, external cortex of the inferior colliculus. PAG and ICe was shaped by dashed lines. **b** A schematic diagram for *Tcf7l2* KO in PAG by AAV-Syn-mCherry-Cre injection. The AAV viral particles were injected into PAG of *Tcf7l2 exon11 fl*/*fl* mouse at P17 and USVs were measured at 3-month of age. The Cre expression was driven by a neuronal Syn1 promoter. AAV-Syn-mCherry served as a control. **c** Immunostaining results show that *Tcf7l2* was conditional KO by AAV-Syn-mCherry-Cre in PAG region. **d**–**g** Key features of USVs measured from *Tcf7l2* conditional KO mice that were injected with virus at P17. **h** Summary of GSEA analysis in PAGs at P7 between *exon11 fl*/*fl* and *exon11 fl*/*fl*;*Vglut2*-*Cre*/+ mice. NES, normalized enrichment score. **i**, **j** Representative mEPSC and mIPSC trace recording from LPAG of + /- and + /+ mice at one-month of age and statistic analysis of the amplitudes and frequencies. In **d**, **e**, **f** and **g**, the value are presented as mean ± SD. N.S., no significant difference, *p < 0.05, **p < 0.01, ***p < 0.001, *t*-test or ANOVA, SPSS. In **d**–**g**, mCherry (*n* = 6); mCherry-Cre (*n* = 12); in **i**, **j**, for mEPSC, +/+, *n* = 21/3; +/-, *n* = 23/3; for mIPSC, +/+, *n* = 22/4; +/-, *n* = 29/3.

Although *Tcf7l2* expression is concentrated in mouse thalamus and midbrain (Fig. S8), thalamic expression of *Tcf7l2* does not contribute to mouse USV production (Fig. 4a), prompting us to hypothesize that *Tcf7l2* expression in PAG is responsible for mouse USV production. Indeed, we found that TCF7L2 is expressed in dorsal and lateral but not ventral PAG (Fig. 5a) and that TCF7L2-positive cells were also positive for *Vglut2-Cre*-driven RPL22-HA expression (Fig. 5a), indicating that TCF7L2-positive cells in PAG are excitatory neurons.

Although TCF7L2 expression is present in the embryonic mouse brain from E12.5, and high in the midbrain between P0 and P7, upon maternal deprivation, pup USV production peaks at P5 or P7 (Fig. S1). To examine whether midbrain *Tcf7l2* expression is responsible for USV production, we injected AAV-mCherry or AAV-mCherry-Cre into the midbrain of *exon11 fl*/*fl* pups at P0 and measured USV at P7 (Fig. S10a, c). We found that the injection of AAV-mCherry-Cre but not AAV-mCherry removed exon 11 (Fig. S10b) in the midbrain as early as P3, three days after the injection, and significantly reduced both short and long TCF7L2 in the midbrain at P7 (Fig. S10d, e). In our USV production analysis, we only included pups with mCherry fluorescence at PAG, and found that, relative to AAV-mCherry injection, the AAV-mCherry-Cre injection significantly decreased syllable numbers but not peak amplitude, syllable duration, and mean frequency (Fig. S10f). We also injected these AAV viral particles into *exon11 fl*/*fl* mouse PAG region at P17 and 2-month of age and measured USV features at 3-month of age (Figs. 5b–g and S11). Both AAV injections at P17 and 2-month achieved more precise AAV infections and TCF7L2 KO at PAG than that at P0 (Figs. 5b, c and S11a, b). However, only P17 but not 2-month AAV-mCherry-Cre injections significantly decreased syllable numbers and peak amplitude, compared to that of AAV-mCherry control injections (Figs. 5d–g and S11c–f). Alternatively, we injected AAV-mCherry-Cre to PAG region of *fx/+* mice at 2-month of age (Fig. S12a). Indeed, the injection partially recovered TCF7L2 expression level in *fx/+* mouse in comparison with that of AAV-mCherry (Fig. S12b, c); however, the injection did not rescue USV abnormalities (Fig. S12d–g). These data suggest that early developmental expression of *Tcf7l2* in PAG is required for pup USV production and adult vocal communication.

### Decrease of synaptic transmission in lateral PAG with loss of TCF7L2

To understand the molecular basis underlying TCF7L2 loss-associated mouse USV impairment, we performed RNA profiling of the PAG region in P7 *exon11 fl*/*fl*; *Vglut2*-*Cre*/+ mice, in which both short and long TCF7L2 are depleted in *Vglut2*-positive neurons (Fig. 4b). Gene Set Enrichment Analysis (GSEA) revealed that several biological pathways were altered in *exon11 fl*/*fl*; *Vglut2*-*Cre*/+ pups relative to *exon11 fl*/*fl* pups (Fig. 5h). Specifically, gene sets involved in neuron projection membrane (normalized enriched score, NES = −2.11, *p* < 0.001), potassium channel activity (NES = −1.92, *p* < 0.001), and synaptic vesicle priming (NES = −1.97, *p* < 0.01) were significantly downregulated in the cKO PAG region, suggesting impaired neuronal function upon depletion of TCF7L2 in the *Vglut2*-positive neurons (Fig. S13a–c). Amongst upregulated gene programs we found genes involved in SMAD signaling (NES = 1.92, *p* < 0.001) and sugar membrane transporter activity (NES = 1.91, *p* < 0.001), suggesting that both BMP/SMAD signaling and glucose metabolism is altered in the cKO PAG (Fig. S13d, e).

To examine whether loss of TCF7L2 indeed causes PAG neuronal dysfunction, we performed whole cell current clamp of the lateral PAG (LPAG) region in acute slices from one-month old *Tcf7l2*^*Y337H/+*^ and *Tcf7l2*^*+/-*^ mice [3]. Overall, haploinsufficiency of *Tcf7l2* did not significantly change neuron excitability nor spike features in LPAG neurons (Fig. S14). However, the miniature excitatory postsynaptic current (mEPSC) frequency and miniature inhibitory postsynaptic current (mIPSC) amplitude were significantly decreased in the *Tcf7l2*^*+/-*^ LPAG neurons relative to + /+ animals (Fig. 5i, j). Taken together, our data suggests that haploinsufficiency of *Tcf7l2* alters neuronal gene expression profiling in a way that impairs LPAG neuron synaptic transmission.

### Full-length of TCF7L2 in *Vglu2*-positive neuron is required for mouse USV production

The TCF7L2 HMG domain is encoded by *Tcf7l2* exon 10 and 11. Because our anti-TCF7L2 antibody was generated from synthetic polypeptide corresponding to sequences in the HMG, and detects both high and low molecular weight bands (Fig. S8e), we conclude that both the long and short form of TCF7L2 contains the HMG region. Indeed, both two-nucleotide deletion in exon 10 (+/-) and conditional removal of exon 11 in *Vglut2*-positive neurons (*exon11 fl*/+;*Vglut2*-*Cre*/+) lead to reduction of both short and long TCF7L2 proteins (Figs. 4b and S5c) and impaired USV (Figs. 4a and S5d, e).

**Fig. 6:**
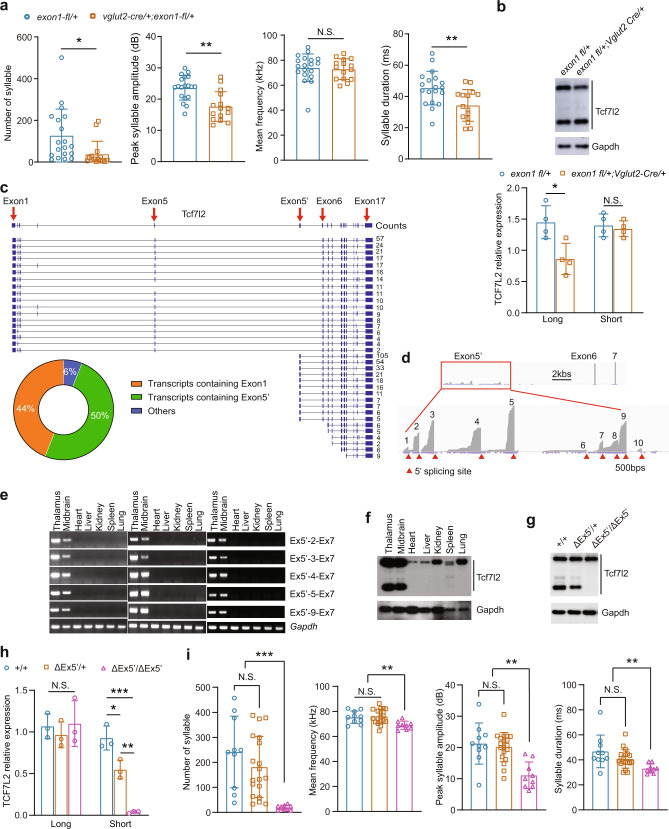
Both flTCF7L2 and dnTCF7L2 are required for mouse USVs. **a** USV measurement in *exon1 fl*/+;*Vglut2*-*Cre*/+ (*n* = 15) at P7. The *exon1 fl*/+ mice (*n* = 19) served as controls. **b** The expression level of TCF7L2 in the midbrain of *exon1 fl*/+;*Vglut2*-*Cre*/+ (*n* = 4) and *exon1 fl*/+ (*n* = 4) mice at P7. GAPDH, loading control. **c** Different isoforms of *Tcf7l2* in mouse brain was identified by nanopore sequencing and the isoform percentages were illustrated. **d** IGV visualization of 10 alternative exon 5’. Red triangles illustrate the 5’ splicing sites of these alternative exon 5’. **e** Transcripts containing exon 5’ were detected in neuronal but not non-neuronal tissues by RT-PCR at P7, 1.5-month, and 3-month-old mice. **f** DnTCF7L2 are expressed in neuronal but not non-neuronal tissues at P7. Expression of dnTCF7L2 in the midbrains in the indicated genotypes at P7 (**g**). The data summary was shown in (**h**) (*n* = 3). **i** USV measurement in the indicated genotypes at P7. In a, b, h, and i, the value are presented as mean ± SD. N.S., no significant difference, *p < 0.05, **p < 0.01, ***p < 0.001, *t*-test or ANOVA, SPSS. In i, +/+ (*n* = 10), *Tcf7l2*^*ΔEx5’/+*^ (*n* = 19), *Tcf7l2*^*ΔEx5’/ΔEx5’*^ (*n* = 9).

Next, we asked which form of TCF7L2 contributes to mouse USV production. To this end, we imported a previously reported *Tcf7l2* exon1 flox allele (*Tcf7l2 exon1 fl*), in which the CBD-encoding exon 1 is flanked by two loxP sites [51]. We found that conditional removal of exon1 in *Vglut2*-positive neurons (*exon1 fl*/+;*Vglut2*-*Cre*/+) significantly impaired pup USVs compared to *exon1 fl*/+ controls (Fig. 6a) and significantly reduced the long but not the short TCF7L2 form in the midbrain (Fig. 6b), indicating that flTCF7L2 is required for mouse USV production.

### Brain-specific dnTCF7L2 is required for mouse vocalization

Our nanopore sequencing, a long-read sequencing technology [52], revealed that about 44% of *Tcf7l2* transcripts in mouse brain contain exon1 (Fig. 6c). However, of all transcripts, about 50% contain exon 5’ and the following exons but not upstream exon 1–5, indicating that TCF7L2^short^ is dnTCF7L2 without the CBD [32, 33, 35, 36]. When we took a closer look at exon 5’, we noticed that at least 10 alternative exon 5’ with 5’ splicing sites are expressed in mouse brain (Fig. 6d). Among these 5’ exons, exon 5’−2, −3, −4, −5, and −9 were covered by more nanopore reads. Indeed, our RT-PCR analysis confirmed expression of these transcripts in midbrain and thalamus at P7, 1.5-, and 3-month of age (Fig. 6e). However, the transcripts were absent in non-neuronal tissues, including heart, liver, kidney, spleen, and lung, suggesting that these transcripts are specific to neuronal tissue. Indeed, dnTCF7L2 proteins were detected in thalamus and midbrain but not in non-neuronal tissues (Fig. 6f).

To validate that dnTCF7L2 is translated from exon 5’-containing transcripts, we removed exon 5’−2–9 by Crispr/Cas9-mediated genome editing (Fig. S15a) and verified that expression of transcripts containing exon 5’−2, −3, −4, −5, and −9 was not detected in thalamus and midbrain in exon 5’ KO mice (ΔEx5’/ΔEx5’) (Fig. S15b). Levels of flTCF7L2 had not changed relative to + */+* mice, however dnTCF7L2 significant reduced in *Tcf7l2*^*ΔEx5’/+*^ mice and could not be detected in *Tcf7l2*^*ΔEx5’/ΔEx5’*^ mice (Fig. 6g, h), suggesting that the brain-specific dnTCF7L2 is indeed translated from these exon 5’-containing transcripts. Adult midbrain flTCF7L2 and dnTCF7L2 (1.5- and 5-month of age) were significantly lower than that of P7, but the ratio of flTCF7L2/dnTCF7L2 shows no significant change during development (Fig. S16). Importantly, we found severe USV abnormalities in *Tcf7l2*^*ΔEx5’/ΔEx5’*^ but not *Tcf7l2*^*ΔEx5’/+*^ and + */+* pups (Fig. 6i), indicating that brain-specific dnTCF7L2 is required for mouse USV production.

### Mice carrying disease-associated mutations produce less USVs and *Tcf7l2*^*+/-*^ mice display normal performance in three-chamber and stereotyped behavior tests

**Fig. 7:**
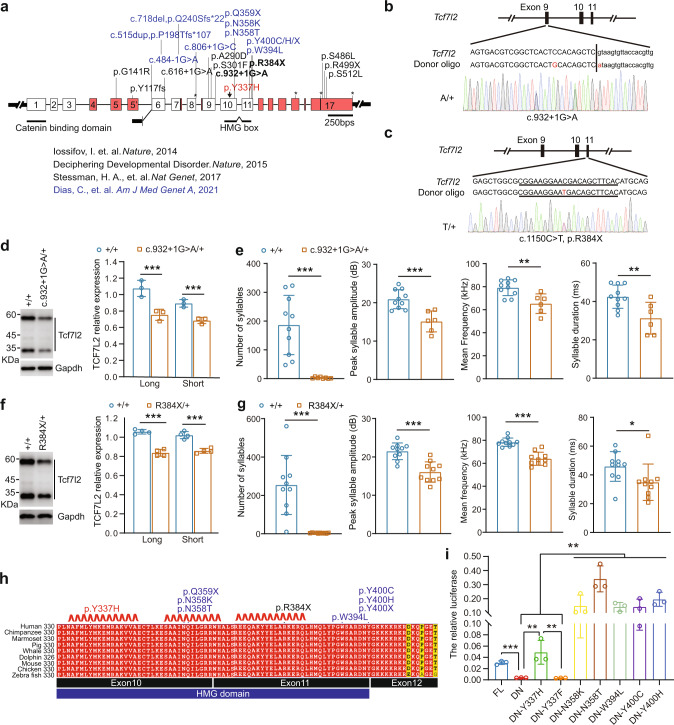
Disease-associated mutations impairs mouse USVs by relieving the transcriptional repression of TCF7L2. **a** Gene structure of *TCF7L2* and human mutations found in patients (PMID: 25363768, 25533962, 28191889, and 34003604). **b**–**e** Generation of *Tcf7l2* knockin mouse lines carrying disease-associated mutations. TCF7L2 expression in P7 midbrains with indicated genotypes (**d**, **f**). **e**, **g** The syllable numbers, mean frequency, syllable duration, and peak syllable amplitude measured at P7 with indicated genotypes. **h** Protein sequence alignment of TCF7L2 HMG domain. Human TCF7L2 (NM_001198528) were employed for positioning. Disease-associated mutations include: 1) our ENU-induced Y337H mutation colored in red; 2) mutations described in previous studies are colored in black (PMID: 25363768) and blue (PMID:34003604), respectively. **i** Transcriptional repression of dnTCF7L2 (DN), which lacks of CBD (beta-catenin binding domain), was relieved by the disease-associated mutations shown in h. Previously reported TOPFlash luciferase reporter (PMID: 9065401) was employed for the measurement. FL, full-length TCF7L2. Renilla luciferase activity was used for normalization. The value are presented as mean ± SD. N.S., no significant difference, **p < 0.01, ***p < 0.001, *t*-test or ANOVA, SPSS. In **d**, **i**, *n* = 3; in **f**, *n* = 4; in **e**, **g**, +/+ (*n* = 10), *Tcf7l2*^*c.932+1G>A/+*^ (*n* = 8), and *Tcf7l2*^*R384X/+*^ (*n* = 10).

Specific *TCF7L2* mutations have been associated with development disorders (DD) and autism spectrum disorder (ASD) [18–21] (Figs. 7a, S17, and Table S1). To provide genetic causality of *Tcf7l2* mutations for disruption of mammal vocalization, we selected two, c.932 + 1 G > A and c.1150 C > T (R384X), to generate knock-in mouse lines (Fig. 7b, c). The c.932 + 1 G > A mutation alters the 5’ splicing site (GT) of *Tcf7l2* exon 9 to AT, potentially preventing correct splicing from exon 9 to exon 10, the latter of which encodes part of the HMG domain. The c.1150 C > T (R384X) mutation introduces a premature termination codon in *TCF7L2* exon 11 (which also encodes the HMG domain) (Fig. 7a), and we therefore speculated that both mutant transcripts would be targeted by nonsense mediated decay for degradation. Indeed, we found that midbrain expression of both flTCF7L2 and dnTCF7L2 in mice heterozygous for the two disease-associated mutations (c.932 + 1 G > A and R384X) was reduced to half of that of wildtype littermates (Fig. 7d, e), suggesting that the two disease-associated mutations are loss-of-function alleles.

Similar to the *Tcf7l2*^*Y337H/+*^ and *Tcf7l2*^*+/-*^ mice, the *Tcf7l2*^*c.932+1G>A/+*^ and *Tcf7l2*^*R384X*^ pups generated fewer syllable numbers, lower peak amplitude, reduced mean frequency, and shorter duration compared to control mice (Fig. 7f, g). Therefore, we conclude that the two *Tcf7l2* disease-associated mutations are likely loss-of-function mutations and impair mouse USV production in a haploinsufficient manner, like the ENU-induced Y337H and *Tcf7l2* KO alleles.

To examine whether deficiency of *Tcf7l2* leads to other phenotype defects, we performed three-chamber test in *Tcf7l2*^*Y337H/+*^ and *Tcf7l2*^*+/-*^ mice. Like wildtype mice, *Tcf7l2*^*Y337H/+*^ and *Tcf7l2*^*+/-*^ spent significantly more sniffing time towards their conspecific than object (Fig. S18a–d) and social preference indexes of *Tcf7l2*^*Y337H/+*^ and *Tcf7l2*^*+/-*^ relative to control mice were comparable, suggesting that *Tcf7l2* haploinsufficiency does not impair social ability. Then we next assessed repetitive behaviors through a marble burying test and estimated self-grooming, we found that *Tcf7l2*^*Y337H/+*^ and *Tcf7l2*^*+/-*^ mice buried similar percentage of marbles and spent similar self-grooming time relative to + */+* mice (Fig. S18e, f). Therefore, we conclude that mice heterozygous for Y337H or KO allele have normal performance in three-chamber and stereotyped behavior tests.

### The disease-associated non-synonymous mutations in HMG domain relieve transcriptional repression of dnTCF7L2

Besides our ENU-induced Y337H mutation, many disease-associated mutations are found in the HMG domain, which is highly conserved from fish to human (Figs. 7h and S17). These mutations include three stop-gain mutations (Q359X, R384X, and Y400X) and five non-synonymous mutations (N358K, N358T, W394L, Y400H, and Y400C). Here, we found that knock-in of the R384X mutation leads to severe USV abnormalities in mouse (Fig. 7f, g), suggesting that the other two stop-gain mutations are likely dysfunctional mutations. Indeed, patients with the Q359X and Y400X mutations displayed developmental disorders, as well as that with the 5 non-synonymous mutations (Table S1).

Because both flTCF7L2 and dnTCF7L2, the latter of which lacks the transcriptionally active domain, are required for vocal production (Fig. 6), we hypothesized that a transcriptional repression mechanism of TCF7L2 is crucial for the complex trait. To this end, we expressed flTCF7L2 and dnTCF7L2 in HEK293 cells and measured their transcriptional activities with an established TCF reporter system (TOPFlash) [53]. Consistent with previous studies [36, 53], the transcriptional activation and repression was achieved by expression of flTCF7L2 (FL) and dnTCF7L2 (DN), respectively (Fig. 7i). However, we found that the repression effect was abolished by Y337H (DN-Y337H) but restored by Y337F (DN-Y337F), indicating that the DNA-binding ability of TCF7L2 is required for the effect. Like Y337H, five disease-associated non-synonymous mutations (DN-N358K, DN-N358T, DN-W394L, DN-Y400H, and DN-Y400C) relieved the transcriptional repression of dnTCF7L2 (Fig. 7i). Therefore, our data is most consistent with a model in which the disease-associated mutations in HMG domain, like Y337H, relieve the transcriptional repression of TCF7L2, which in turn leads to vocal abnormalities in patients.

## Discussion

Here, through the use of a battery of genetic mouse models, we show that TCF7L2 acts as a molecular switch in midbrain PAG *Vglut2*-positive neurons to control mouse USV production and syllable complexity through its DNA binding but not transcription activation domain. We summarized the *Tcf7l2* lines we generated or employed and their corresponding phenotypes in Table S2. Previously, transcriptional repression mechanisms and Wnt/β-catenin signaling activation independent roles of TCF7L2 have been documented during oligodendrocyte differentiation and brain development [35, 36, 54–57]. In addition, transcriptional repression of BMP4/SMAD signaling by TCF7L2 has been linked to oligodendrocyte differentiation [55]. In agreement with these findings, we observed that expressions of genes involved in BMP/SMAD but not Wnt/β-catenin signaling are upregulated in *Tcf7l2* cKO PAG (Fig. S13d). Given that dnTCF7L2 inhibits the Wnt/β-catenin signaling cascade [32, 33, 35, 36] and is required for the vocal control (Fig. 6i), we argue that a transcriptional repression mechanism mediated by TCF7L2 is crucial for control of the complex vocalization trait. Indeed, our data demonstrates that like Y337H, patient mutations associated with developmental disorders relieve the transcriptional repression of TCF7L2 (Fig. 7i).

As we demonstrate that loss of TCF7L2 and the Y337H mutation in the HMG domain result in vocalization abnormalities in mice, it is important to note that patients carrying *de novo* genetic variants, including loss-of-function mutations and non-synonymous mutations in HMG domain similar to what we reported here, display developmental disorder [18–21]. Like Y337H, human non-synonymous mutations in HMG domain we examined may affect TCF7L2 DNA-binding capability (Fig. 7i). Because mice heterozygous for Y337H or KO allele displayed similar RNA profiling in thalamus and midbrain and vocal abnormalities (Fig. S7), we argue the non-synonymous mutations in HMG may lead to the disease through a loss-of-function mechanism as Y337H does. Clearly, the mice heterozygous for human c.932 + 1 G > A or R384X mutations are loss-of-function alleles (Fig. 7d, f). Importantly, our knock-in mice with such disease-associated mutations display severe USV abnormalities, providing genetic causality of those patient mutations for impairment of the complex trait. These results also indicate that the cellular and molecular mechanisms we identify here are most likely shared by mouse and human. Consistent with less ASD penetration in the patients [21], the mice heterozygous for Y337H or KO allele have normal performance in the social and stereotyped behavior tests (Fig. S18).

At neural circuit level, two separate pathways control vocalization: 1) from anterior cingulate cortex-PAG-brain stem, which is considered as a key pathway for innate vocalization [5]; 2) from motor cortex-reticular formation-phonatory motoneurons, which is believed for human and songbird vocal learning process but not for the species that produce only innate vocalization [5, 58]. In fact, people with PAG damages are not able to speak [1, 5, 6]. Given that PAG functions as an organizing node for mammal vocalization in the brain [1–3], the impaired synaptic transmissions in LPAG we detected here may be the consequence of TCF7L2 loss of function, thereby explaining the mechanisms of vocalization deficits (Fig. 5i, j). In addition to PAG’s role as a central vocalization node, *Esr1*-positive neurons in the ventromedial hypothalamus and POA and *Vgat*-positive inhibitory neurons in amygdala (Amg) act upstream of PAG-USV neurons to contribute to mouse USV production and persistence [44–47]. We demonstrate that expression of TCF7L2 in *Vglut2*-positive neurons is essential for mouse USV production, excluding the possibility that expression of TCF7L2 in *Vgat*-positive neurons in POA, Amg, and PAG contribute to the processes. By removing *Tcf7l2* in *Esr1*-positive and *ChAT*-positive neurons, we further exclude the possibilities that *Esr-1*-positive neurons in VMH and POA and *ChAT*-positive neurons in medial habenula and spinal cord motor neurons participate in the processes (Figs. 4a and S19). Given that *TCF7L2* has been associated with metabolic disorders [16] and glucose metabolism is affected in PAG by loss of TCF7L2 in *Vglut2*-positive neurons (Fig. S13e), metabolic status may influence PAG-USV neuron activities and contribute to mammal vocal production and performance. By nanopore sequencing, we demonstrate that *Tcf7l2* undergoes multiple alternative splicing, including at least 10 different exon5’ usages for transcripts encoding dnTCF7L2, in midbrain (Fig. 6d). In future work, it will be intriguing to investigate *TCF7L2* alternative splicing patterns across different species to syllable complexity and human language evolution.

## Material and methods

### Animals

Experimental procedures were approved by IACUC (Institutional Animal Care and Use Committee) of Tsinghua University. All the mice described in this research were C57BL/6 J background and housed in room at 22–26 °C in 12/12-hour light/dark cycle with sterile pellet food and water ad libitum. The *Vglut2*-cre mouse (# 016963, Jackson Laboratory) was gifted from Dr. Minmin Luo at NIBS. *Olig3*-cre [43] was kindly provided by Dr. Songhai Shi; *Esr1*-cre (#017911, Jax) was gifted from Dr. Xiaohong Xu; *Tcf7l2* exon11 floxed mouse was gifted from Dr. Richard Lu; *Nestin*-cre (#003771, Jax), *ChAT*-cre (#006410, Jax), and *Tcf7l2* exon1 floxed mouse (#031436, Jax) were imported from The Jackson Laboratory.

### Forward genetics screening and mutant candidate identification

Male C57BL/6 J mice were treated with ENU (80–110 mg/kg B. W.) for three consecutive weeks by intraperitoneal injection at the age of 12 weeks according previously reported [59]. The ENU-treated males were crossed with C57BL/6 J females and the resulting G1 pups were subjected to USV measurement at P5 with maternal deprivation in a 5-minute interval by a commercial USV detector (Med Associates Inc). The USV impaired G1 mice (≤5 in the 5-minute interval we measured) were crossed to C57BL/6 J and the recurrence of USV impairment in G2 and G3 offsprings was used to establish the family pedigree and the genomic DNA was applied for whole exome sequencing. Mouse genomic DNA from USV affected and unaffected mice in the family was sheared into 200–500 bp fragments and captured by SeqCap EZ probe pool (Roche). The libraries were sequenced through HiSeq and the reads were aligned to GRCm38 mouse reference genome. The mutant candidates were selected by previously described criteria [59].

### Knockout and FLEX (fx) conditional knockin mouse generation by CRISPR/Cas9 technology

All the sgRNAs were designed by Cas-Designer (http://www.rgenome.net/cas-designer/). Cas9 mRNA and sgRNAs were synthetized and purified by HiScribe™ T7 High Yield RNA Synthesis Kit (E2040S, NEB) and Purification of Synthesized RNA (E2040, NEB), respectively. Mixed Cas9 mRNA, sgRNAs, and/or donor DNA were injected into C57BL/6 J mouse zygotes at the pronuclei stage. The injected eggs were transferred to into uterus of pseudopregnant ICR females. For generation of *Foxp2* Δ5 allele, the sgRNA: CCATACGAAGGCGACATTCAGAC; for *Tcf7l2* Δ2 allele, the sgRNA: TATGAAAGAGATGAGAGCGA; for *Tcf7l2 fx*, the sgRNAs: CCAGTGATTGAAACTGACAC, CCAGTGTCAGTTTCAATCAC, GATTCCACCCAGGCAGAAAA, TAACGGGTTTGATTCCACCC; for *Tcf7l2* ΔEx5’, the sgRNAs: ATGAATGATCGGGTTCTTTG, GAAGAACTGAGACAAAATGT, CCACAGAGGACTGCTACCAT, CCTATGGTAGCAGTCCTCTG. The donor DNA for *Tcf7l2 fx* mouse include the inverted exon9 and exon10 and their surrounding sequences flanked by two pairs of incompatible lox sites (loxP and lox2722) and homology arms (~3 kb).

### Immunofluorescence, RNA in situ hybridization, and western blot

Mice was anesthetized and transcardially perfused with cold PBS and 4% PFA. Brains were post fixed and dehydrated with 30% sucrose and sectioned to 20 μm. The sections were blocked and permeabilized with 3% BSA and 0.3% Triton X-100 in PBS, and then incubated with the primary antibody overnight at 4 °C. Sections were washed with PBST three times and incubated with the secondary antibody. The antibodies: rabbit anti-TCF7L2 (Cell Signaling Technology, #2569, 1:200), mouse anti-HA (Abcam, ab18181, 1:500), donkey anti-mouse IgG secondary antibody (Alexa Fluor 555, Thermo Fisher Scientific, A31570, 1:1000), donkey anti-mouse IgG secondary antibody (Alexa Fluor 488, Thermo Fisher Scientific, A32766, 1:1000). RNA in situ hybridization was performed according to RNAscope manual. For western blot, tissues were homogenized in RIPA buffer (50 mM Tris-HCl, pH 8.0, 150 mM NaCl, 0.25% sodium deoxycholate, 0.1% SDS, and 1% NP-40) supplemented with complete protease inhibitor mixture (Roche). Lysates were centrifuged at 12000 x g for 10 min at 4 °C. The membranes were incubated with the primary antibodies overnight at 4 °C, then washed with PBST and incubated with HRP-conjugated secondary antibodies. Intensities of blot bands were calculated by Image J and subtracted by background. The relative expression was normalized to loading control GAPDH.

### Protein expression and purification and electrophoresis mobility shift assay (EMSA)

The coding sequences of wild type and mutant HMG domain were cloned into pMAL-cRI plasmid with MBP (Maltose Binding Protein) tag. The expression of recombinant HMG proteins was induced by 0.3 mM IPTG in BL21 at 30 °C. Cells were lysed by ultrasonic homogenizer. After centrifugation, the recombinant proteins were purified by amylose resin (#E8022S, New England Biolabs) according the manual instructions. EMSA was performed and HMG-binding sequences (5’-tagacgtagggcaccctttgaagctctccctcga) were synthesized as previously described [60]. Briefly, biotin-labeled probe was incubated with different HMG proteins in EMSA buffer (50 mM NaCl, 20 mM Tris-HCl, 1 mM DTT, 1 mM MgCl_2_, 5% Glycerol) for 1 h at 25 °C. Then, the samples were separated with 6% native PAGE, and transferred to nylon membranes. After UV crosslinking, the membrane was incubated with HRP-conjugated Streptavidin. Finally, the signal was visualized by GE imaging system.

### Luciferase assay

The plasmid of TOPflash, pCMV-Flag-β-catenin, and pCMV-Renilla together with each dnTCF7L2 point mutation (DN-Y337H, DN-Y337F, DN-N358K, DN-N358T, DN-W394L, DN-Y400H, and DN-Y400C) were transfected into HEK293T cells in six-well plates. Cell lysis were harvested 12 h after transfection for luciferase analysis according to the manual instruction (#RG027, Beyotime). The value of luciferase was measured by VARIOSKAN FLASH (Thermo).

### Reverse Transcription PCR (RT-PCR), real-time PCR, RNA-Seq, and nanopore sequencing

For RT-PCR, total RNA was isolated by TRIzol reagent (Thermo Fisher Scientific). RNA was treated with DNase and cDNA was synthesized by M-MLV Reverse Transcriptase (Promega). For real-time PCR, the reaction was performed with 2 x Hieff qPCR SYBR Green Master Mix (Yeasen) and detected by LightCycle 480 Real-Time PCR machine (Roche). For RNA-seq, RNA sample library preparation was performed according to TruSeq mRNA-seq Stranded v2 Kit (Illumina) manual instruction. The library was sequenced using Illumina Hiseq platform. Reads were aligned to GRCm38 mouse reference. HTSeq was used to quantify the aligned reads [61]. Reads normalization and differential gene expression analysis were performed using DESeq2 [62]. Normalized reads were subjected to Gene Set Enrichment Analysis (GSEA) [63]. For nanopore sequencing, RNA library was constructed following manual instruction (Oxford Nanopore Technologies) and the sequencing was conducted using Oxford Nanopore Technology’s MinION. Long reads were aligned to GRCm38 mouse reference using minimap2 [64]. Isoform definition and quantification were achieved by FLAIR [65]. Reads were visualized by Integrative Genomics Viewer (IGV) [66].

### AAV preparation and injection

AAV2/9 Syn- mCherry-Cre and Syn-mCherry were purchased from OBiO (Shanghai). After born within 6 h, the mice were anesthetized with ice and the head was fixed by plasticine in stereotaxic apparatus. AAV virus (60 nl) were infused by glass micropipettes at a rate of 50 nl/min bilaterally in PAG region (−0.8 mm AP from Lambda site; ± 0.35 mm ML; −1.85 mm DV). The injected mice were put on warm blanket to recover 1 h at 37 °C. For adult and P17 mouse injection, the mice were anesthetized with isoflurane and the head was fixed by plasticine in stereotaxic apparatus. AAV virus (80 nl) were infused by glass micropipettes at a rate of 50 nl/min bilaterally in PAG region (−4.65 mm AP from Bregma site; ± 0.55 mm ML; −2.35 mm DV).

### Electrophysiology

Electrophysiological recording was performed with MultiClamp 700B amplifier (Molecular Devices, USA) as previously described [67]. Mice (P25–P30) were anesthetized and transcardially perfused with ice-cold oxygenated cutting aCSF, containing (in mM) 125 sucrose, 1.25 NaH_2_PO_4_, 2 CaCl_2_, 3 KCl, 2 MgSO_4_, 26 NaHCO_3_, 1.3 sodium ascorbate, and 0.6 sodium pyruvate. PAG slices (300 μm) were prepared and recovered in normal aCSF, containing (in mM): 125 NaCl, 1.25 NaH_2_PO_4_, 2 CaC_l2_, 3 KCl, 2 MgSO_4_, 26 NaHCO_3_, 1.3 sodium ascorbate, and 0.6 sodium pyruvate, for 25 min at 34 °C, and then maintained at room temperature for 1 h before recording. For mEPSC, slices were transferred into recording chamber perfused with aCSF in the presence of 1 μM tetrodotoxin and 50 μM picrotoxin. Recording micropipettes were prepared by Sutter P97 puller with a resistance of 3–5 MΩ and filled with internal solution, containing (in mM): 130 potassium-gluconate, 20 KCl, 10 HEPES, 10 Na_2_HPO_4_, 4 Mg_2_ATP, 0.3 Na_2_GTP, and 0.2 mM EGTA (pH 7.35, 299 mOsm). For mIPSC, the slices were bathed in aCSF with 10 μM CNQX and 50 μM AP5 and recording internal solution containing (in mM): 60 CsCl, 2 MgCl_2_, 10 HEPES, 10 Na_2_HPO_4_, 4 Mg_2_ATP, 0.3 Na_2_GTP, and 0.2 mM EGTA (pH 7.35, 299 mOsm). For neuron excitability measurement, action potential firing was recorded under current clamp mode with pipette filled with the internal solution used for mEPSC. Neuron was injected with currents from 0 to 300 pA with an increment of 50 pA.

### Mouse behavior analysis

Open field, rotarods, marble buried test, and self-grooming test were performed in a proper order from two-month of age. Adult vocalization and three-chamber test were performed at three-month of age. We performed animal behavior tests in a double-blind manner. The sample size was determined based on the previous similar studies in the field.

#### Open field

Two-month old mice were placed in the box (50 × 50 × 50 cm) and freely explored for 30 min. Total travel distance and time spent in the center area were calculated by TopScan (CleverSys Inc.) behavior analysis software.

#### Rotarod performance test

Two-month old mice were put on the accelerating rotarod with speed from 4 to 40 rpm in 5-minute and three consecutive trials per day for 3 days. A 20-minute break was set in between each trial. The fall time from the spindle was auto-calculated by the system (Med Associates Inc.).

#### Ultrasonic vocalization (USV) measurement

For pup vocalization, pup was isolated from their littermates and mom and placed into a glass cup in a soundproof box. USVs were recorded by ultrasound microphone (Avisoft CM16/CMPA) for 5 min. For adult mouse USV measurement, 3-month-old males were single caged for 5 days before the test. After the tested male was habituated in the soundproof box for 5 min, live or anesthetized virgin female was placed into the box and USVs were recorded. For analysis of USVs, syllable numbers, peak syllable amplitude, mean frequency, and syllable duration were measured by MUPET [38]. We set the length of syllable duration from 8 ms to 200 ms. The minimum syllable distance is not shorter than 5 ms, if less than 5 ms the software will recognize as one syllable. The minimum syllable peak amplitude is set as −25. The thresh old of syllable frequency is from 35 kHz to 110 kHz. Syllable repertoire composition was classified into 4 categories: 1) simple syllables “s” only containing one note; 2) downward syllables “d” containing two notes that from a higher frequency note jumps to a lower frequency note; 3) upward “u” containing two notes that from a lower frequency note jumps to a higher frequency note; 4) multiple syllables “m” containing three or more notes by Mouse Song Analyzed v1.3 [68]. We termed syllable transition as one syllable to another syllable, like “s” to “s”, “s” to “d”, “m” to “s”, and so on. Syllable transitions of conditional probability for each context were analyzed by a customized script generated in Microsoft Excel [68].

#### Three-chamber social ability test

A rectangular box (60 length × 40 width × 25 height, cm) consisted of three chambers (20 × 40 × 25 cm) side by side. The tested mouse was allowed to habituate the chambers for 5 min and then placed in the center chamber. A stranger mouse (S) was placed in a container in one side of the chamber and an object (O, another container) was placed in the other side. Then the tested mouse was allowed to explore S and O for 10 min and the sniffing time toward S and O was calculated manually. Social preference index was calculated as previously described [69].

#### Female preference test

Female was placed in the three chamber as described above and allowed to explore the chamber for 5 min, and then placed in the center chamber. Wildtype (+/+) mouse was placed in a container in one side chamber and *Tcf7l2*^*Y337H/+*^ or *Tcf7l2*^*+/-*^ mutant mouse was placed in the other side. Then the tested female was allowed to explore the chamber freely for 20 min and the sniffing time toward each mouse was calculated manually. The preference index was measured as (time spent on one side with male)/(time spent on two sides with male).

#### Marbles buried test

Fresh bedding (5 cm) was filled in a standard mouse cage with 20 dark glass marbles (1.5 cm diameter), which were placed on the bedding in a symmetrical 4 × 5 pattern. Each tested mouse was allowed to explore freely for 30 min. The number of buried marbles (>2/3 height of marble covered with bedding) was counted.

#### Self-grooming test

The tested mouse was placed in a standard mouse cage with fresh bedding and videotaped for 20 min. The time of self-grooming behaviors (including rubbing/scratching head, face, and other body parts) were manually accounted.

## Supplementary information

Table S1

Table S2

Supplementary_Figure

Figure_Legends

## References

[1] Nieder A, Mooney R (2020). The neurobiology of innate, volitional and learned vocalizations in mammals and birds. Philos Trans R Soc Lond B Biol Sci.

[2] Jurgens U, Hage SR (2007). On the role of the reticular formation in vocal pattern generation. Behav Brain Res.

[3] Tschida K, Michael V, Takatoh J, Han BX, Zhao S, Sakurai K (2019). A Specialized Neural Circuit Gates Social Vocalizations in the Mouse. Neuron.

[4] Jarvis ED (2019). Evolution of vocal learning and spoken language. Science.

[5] Juergens U (2009). The Neural Control of Vocalization in Mammals: A Review. J Voice.

[6] Esposito A, Demeurisse G, Alberti B, Fabbro F (1999). Complete mutism after midbrain periaqueductal gray lesion. Neuroreport..

[7] Lai CS, Fisher SE, Hurst JA, Vargha-Khadem F, Monaco AP (2001). A forkhead-domain gene is mutated in a severe speech and language disorder. Nature..

[8] Hamdan FF, Daoud H, Rochefort D, Piton A, Gauthier J, Langlois M (2010). De Novo Mutations in FOXP1 in Cases with Intellectual Disability, Autism, and Language Impairment. Am J Hum Genet.

[9] Horn D, Kapeller J, Rivera-Brugues N, Moog U, Lorenz-Depiereux B, Eck S (2010). Identification of FOXP1 deletions in three unrelated patients with mental retardation and significant speech and language deficits. Hum Mutat.

[10] Newbury DF, Monaco AP (2010). Genetic advances in the study of speech and language disorders. Neuron..

[11] Konopka G, Roberts TF (2016). Insights into the Neural and Genetic Basis of Vocal Communication. Cell..

[12] Castellucci GA, McGinley MJ, McCormick DA (2016). Knockout of Foxp2 disrupts vocal development in mice. Sci Rep.

[13] Chabout J, Sarkar A, Patel SR, Radden T, Dunson DB, Fisher SE (2016). A Foxp2 Mutation Implicated in Human Speech Deficits Alters Sequencing of Ultrasonic Vocalizations in Adult Male Mice. Front Behav Neurosci.

[14] Chen YC, Kuo HY, Bornschein U, Takahashi H, Chen SY, Lu KM (2016). Foxp2 controls synaptic wiring of corticostriatal circuits and vocal communication by opposing Mef2c. Nat Neurosci.

[15] Frohlich H, Rafiullah R, Schmitt N, Abele S, Rappold GA (2017). Foxp1 expression is essential for sex-specific murine neonatal ultrasonic vocalization. Hum Mol Genet.

[16] Grant SF, Thorleifsson G, Reynisdottir I, Benediktsson R, Manolescu A, Sainz J (2006). Variant of transcription factor 7-like 2 (TCF7L2) gene confers risk of type 2 diabetes. Nat Genet.

[17] Gonzalez N, Prieto I, Del Puerto-Nevado L, Portal-Nunez S, Ardura JA, Corton M (2017). 2017 update on the relationship between diabetes and colorectal cancer: epidemiology, potential molecular mechanisms and therapeutic implications. Oncotarget.

[18] Iossifov I, O’Roak BJ, Sanders SJ, Ronemus M, Krumm N, Levy D (2014). The contribution of de novo coding mutations to autism spectrum disorder. Nature.

[19] Deciphering Developmental Disorders S. (2015). Large-scale discovery of novel genetic causes of developmental disorders. Nature..

[20] Stessman HA, Xiong B, Coe BP, Wang T, Hoekzema K, Fenckova M (2017). Targeted sequencing identifies 91 neurodevelopmental-disorder risk genes with autism and developmental-disability biases. Nat Genet.

[21] Dias C, Pfundt R, Kleefstra T, Shuurs-Hoeijmakers J, Boon EMJ, van Hagen JM (2021). De novo variants in TCF7L2 are associated with a syndromic neurodevelopmental disorder. Am J Med Genet A.

[22] Bem J, Brozko N, Chakraborty C, Lipiec MA, Kozinski K, Nagalski A (2019). Wnt/beta-catenin signaling in brain development and mental disorders: keeping TCF7L2 in mind. FEBS Lett.

[23] Liu L, Li J, Yan M, Li J, Chen J, Zhang Y (2017). TCF7L2 polymorphisms and the risk of schizophrenia in the Chinese Han population. Oncotarget.

[24] Alkelai A, Greenbaum L, Lupoli S, Kohn Y, Sarner-Kanyas K, Ben-Asher E (2012). Association of the type 2 diabetes mellitus susceptibility gene, TCF7L2, with schizophrenia in an Arab-Israeli family sample. PLoS One.

[25] Hansen T, Ingason A, Djurovic S, Melle I, Fenger M, Gustafsson O (2011). At-risk variant in TCF7L2 for type II diabetes increases risk of schizophrenia. Biol Psychiatry.

[26] Cuellar-Barboza AB, Winham SJ, McElroy SL, Geske JR, Jenkins GD, Colby CL (2016). Accumulating evidence for a role of TCF7L2 variants in bipolar disorder with elevated body mass index. Bipolar Disord.

[27] Lipiec MA, Bem J, Kozinski K, Chakraborty C, Urban-Ciecko J, Zajkowski T, et al. TCF7L2 regulates postmitotic differentiation programmes and excitability patterns in the thalamus. Development. 2020;147:dev190181. 10.1242/dev.190181 PMC7473649 32675279

[28] Duncan A, Heyer MP, Ishikawa M, Caligiuri SPB, Liu XA, Chen Z (2019). Habenular TCF7L2 links nicotine addiction to diabetes. Nature..

[29] Savic D, Distler MG, Sokoloff G, Shanahan NA, Dulawa SC, Palmer AA, et al. Modulation of Tcf7l2 Expression Alters Behavior in Mice. Plos One. 2011;6:e26897. 10.1371/journal.pone.0026897 PMC3203170 22046400

[30] Nusse R, Clevers H (2017). Wnt/beta-Catenin Signaling, Disease, and Emerging Therapeutic Modalities. Cell..

[31] Clevers H, Nusse R (2012). Wnt/beta-catenin signaling and disease. Cell..

[32] Cadigan KM, Waterman ML. TCF/LEFs and Wnt signaling in the nucleus. Cold Spring Harb Perspect Biol. 2012;4:a007906. 10.1101/cshperspect.a007906 PMC3536346 23024173

[33] Hoppler S, Kavanagh CL (2007). Wnt signalling: variety at the core. J Cell Sci.

[34] Daniels DL, Weis WI (2005). Beta-catenin directly displaces Groucho/TLE repressors from Tcf/Lef in Wnt-mediated transcription activation. Nat Struct Mol Biol.

[35] Vacik T, Lemke G (2011). Dominant-negative isoforms of Tcf/Lef proteins in development and disease. Cell Cycle.

[36] Vacik T, Stubbs JL, Lemke G (2011). A novel mechanism for the transcriptional regulation of Wnt signaling in development. Genes Dev.

[37] Love JJ, Li XA, Case DA, Giese K, Grosschedl R, Wright PE (1995). Structural basis for DNA bending by the architectural transcription factor LEF-1. Nature..

[38] Van Segbroeck M, Knoll AT, Levitt P, Narayanan S (2017). MUPET-Mouse Ultrasonic Profile ExTraction: A Signal Processing Tool for Rapid and Unsupervised Analysis of Ultrasonic Vocalizations. Neuron..

[39] Chabout J, Sarkar A, Dunson DB, Jarvis ED (2015). Male mice song syntax depends on social contexts and influences female preferences. Front Behav Neurosci.

[40] Yang M, Loureiro D, Kalikhman D, Crawley JN. Male mice emit distinct ultrasonic vocalizations when the female leaves the social interaction arena. Front Behav Neurosci. 2013;7:159. 10.3389/fnbeh.2013.00159 PMC3832782 24312027

[41] Nagalski A, Irimia M, Szewczyk L, Ferran JL, Misztal K, Kuznicki J (2013). Postnatal isoform switch and protein localization of LEF1 and TCF7L2 transcription factors in cortical, thalamic, and mesencephalic regions of the adult mouse brain. Brain Struct Funct.

[42] van Es JH, Haegebarth A, Kujala P, Itzkovitz S, Koo BK, Boj SF (2012). A critical role for the Wnt effector Tcf4 in adult intestinal homeostatic self-renewal. Mol Cell Biol.

[43] Vue TY, Bluske K, Alishahi A, Yang LL, Koyano-Nakagawa N, Novitch B (2009). Sonic hedgehog signaling controls thalamic progenitor identity and nuclei specification in mice. J Neurosci.

[44] Chen J, Markowitz JE, Lilascharoen V, Taylor S, Sheurpukdi P, Keller JA (2021). Flexible scaling and persistence of social vocal communication. Nature.

[45] Karigo T, Kennedy A, Yang B, Liu M, Tai D, Wahle IA (2021). Distinct hypothalamic control of same- and opposite-sex mounting behaviour in mice. Nature.

[46] Michael V, Goffinet J, Pearson J, Wang F, Tschida K, Mooney R. Circuit and synaptic organization of forebrain-to-midbrain pathways that promote and suppress vocalization. Elife. 2020;9:e63493. 10.7554/eLife.63493 PMC7793624 33372655

[47] Gao SC, Wei YC, Wang SR, Xu XH (2019). Medial Preoptic Area Modulates Courtship Ultrasonic Vocalization in Adult Male Mice. Neurosci Bull.

[48] Arriaga G, Zhou EP, Jarvis ED (2012). Of mice, birds, and men: the mouse ultrasonic song system has some features similar to humans and song-learning birds. PLoS One.

[49] Jurgens U (2002). Neural pathways underlying vocal control. Neurosci Biobehav Rev.

[50] Schnutgen F, Doerflinger N, Calleja C, Wendling O, Chambon P, Ghyselinck NB (2003). A directional strategy for monitoring Cre-mediated recombination at the cellular level in the mouse. Nat Biotechnol.

[51] Angus-Hill ML, Elbert KM, Hidalgo J, Capecchi MR (2011). T-cell factor 4 functions as a tumor suppressor whose disruption modulates colon cell proliferation and tumorigenesis. Proc Natl Acad Sci USA.

[52] Venkatesan BM, Bashir R (2011). Nanopore sensors for nucleic acid analysis. Nat Nanotechnol.

[53] Korinek V, Barker N, Morin PJ, van Wichen D, de Weger R, Kinzler KW (1997). Constitutive transcriptional activation by a beta-catenin-Tcf complex in APC-/- colon carcinoma. Science.

[54] Ye F, Chen Y, Hoang T, Montgomery RL, Zhao XH, Bu H (2009). HDAC1 and HDAC2 regulate oligodendrocyte differentiation by disrupting the beta-catenin-TCF interaction. Nat Neurosci.

[55] Zhang S, Wang Y, Zhu X, Song L, Zhan X, Ma E (2021). The Wnt Effector TCF7l2 Promotes Oligodendroglial Differentiation by Repressing Autocrine BMP4-Mediated Signaling. J Neurosci.

[56] Zhao C, Deng Y, Liu L, Yu K, Zhang L, Wang H (2016). Dual regulatory switch through interactions of Tcf7l2/Tcf4 with stage-specific partners propels oligodendroglial maturation. Nat Commun.

[57] Hammond E, Lang J, Maeda Y, Pleasure D, Angus-Hill M, Xu J (2015). The Wnt effector transcription factor 7-like 2 positively regulates oligodendrocyte differentiation in a manner independent of Wnt/beta-catenin signaling. J Neurosci.

[58] Jarvis ED. Learned birdsong and the neurobiology of human language. Ann N Y Acad Sci. 2004;1016:749–77. 10.1196/annals.1298.038 PMC2485240 15313804

[59] Chen RZ, Cheng X, Tan Y, Chang TC, Lv H, Jia Y (2020). An ENU-induced mutation in Twist1 transactivation domain causes hindlimb polydactyly with complete penetrance and dominant-negatively impairs E2A-dependent transcription. Sci Rep.

[60] Giese K, Amsterdam A, Grosschedl R (1991). DNA-binding properties of the HMG domain of the lymphoid-specific transcriptional regulator LEF-1. Genes Dev.

[61] Anders S, Pyl PT, Huber W (2015). HTSeq-a Python framework to work with high-throughput sequencing data. Bioinformatics..

[62] Love MI, Huber W, Anders S. Moderated estimation of fold change and dispersion for RNA-seq data with DESeq2. Genome Biol. 2014;15:550. 10.1186/s13059-014-0550-8 PMC4302049 25516281

[63] Subramanian A, Tamayo P, Mootha VK, Mukherjee S, Ebert BL, Gillette MA (2005). Gene set enrichment analysis: A knowledge-based approach for interpreting genome-wide expression profiles. Proc Natl Acad Sci USA.

[64] Li H (2018). Minimap2: pairwise alignment for nucleotide sequences. Bioinformatics.

[65] Tang AD, Soulette CM, van Baren MJ, Hart K, Hrabeta-Robinson E, Wu CJ, et al. Full-length transcript characterization of SF3B1 mutation in chronic lymphocytic leukemia reveals downregulation of retained introns. Nat Commun. 2020;11:1438. 10.1038/s41467-020-15171-6 PMC7080807 32188845

[66] Robinson JT, Thorvaldsdottir H, Winckler W, Guttman M, Lander ES, Getz G (2011). Integrative genomics viewer. Nat Biotechnol.

[67] Li Y, Xu J, Liu Y, Zhu J, Liu N, Zeng W (2017). A distinct entorhinal cortex to hippocampal CA1 direct circuit for olfactory associative learning. Nat Neurosci.

[68] Chabout J, Jones-Macopson J, Jarvis ED. Eliciting and Analyzing Male Mouse Ultrasonic Vocalization (USV) Songs. J Vis Exp. 2017;123:54137. 10.3791/54137 PMC5607930 28518074

[69] Dong Z, Chen W, Chen C, Wang H, Cui W, Tan Z (2020). CUL3 Deficiency Causes Social Deficits and Anxiety-like Behaviors by Impairing Excitation-Inhibition Balance through the Promotion of Cap-Dependent Translation. Neuron.

