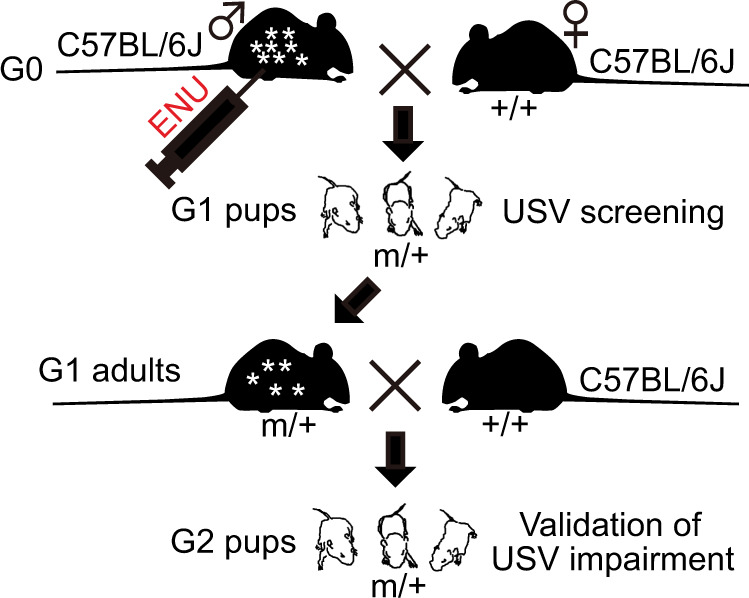# Correction to: TCF7L2 acts as a molecular switch in midbrain to control mammal vocalization through its DNA binding domain but not transcription activation domain

**DOI:** 10.1038/s41380-023-02011-4

**Published:** 2023-03-03

**Authors:** Huihui Qi, Li Luo, Caijing Lu, Runze Chen, Xianyao Zhou, Xiaohui Zhang, Yichang Jia

**Affiliations:** 1 Tsinghua-Peking Joint Center for Life Sciences, Tsinghua University, Beijing, 100084 China; 2 School of Medicine, Tsinghua University, Beijing, 100084 China; 3 IDG/McGovern Institute for Brain Research, Tsinghua University, Beijing, 100084 China; 4 Tsinghua Laboratory of Brain and Intelligence (THBI), Tsinghua University, Beijing, 100084 China; 5 School of Life Sciences, Tsinghua University, Beijing, 100084 China; 6 Key Laboratory of Birth Defects and Related Diseases of Women and Children of Ministry of Education, Sichuan University, Chengdu, China; 7 State Key Laboratory of Cognitive Neuroscience and Learning, IDG/McGovern Institute for Brain Science, Beijing Normal University, Beijing, 100875 China

**Keywords:** Neuroscience, Autism spectrum disorders

Correction to: *Molecular Psychiatry* 10.1038/s41380-023-01993-5, published online 13 February 2023

In Fig. 1a of this article “X” symbol shows error; the Fig. 1a should have appeared as shown below.

The original article has been corrected.