# Supplementary material for: TCF7L2 acts as a molecular switch in midbrain to control mammal vocalization through its DNA binding domain but not transcription activation domain
Source: Mol Psychiatry. 2023 Feb 13;28(4):1703–17. doi: 10.1038/s41380-023-01993-5 (PMC10208975; doi:10.1038/s41380-023-01993-5)
Supplement: Supplementary file 3 — Supplementary_Figure [file 41380_2023_1993_MOESM3_ESM.pdf]

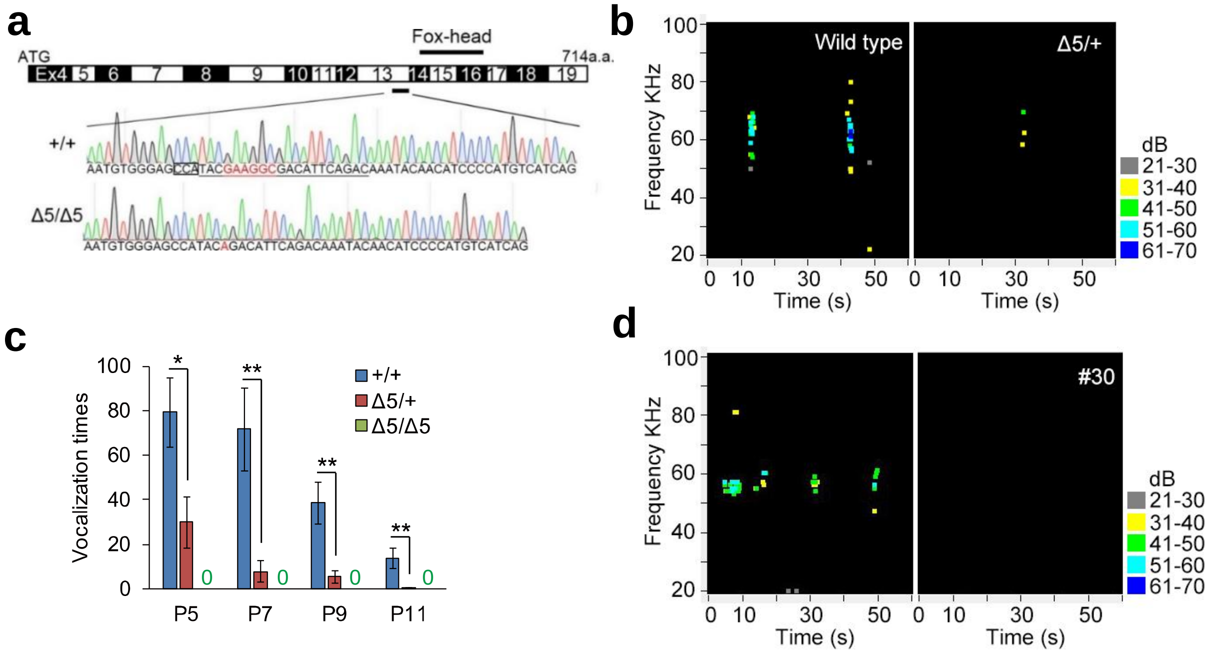
a
Fox-head
ATG
714a.a.
Ex4 5 6 7 8 9 10 11 12 13 14 15 16 17 18 19
+/+
AATGTGGGAGCCATACGAAGGCGACATTCAGACAAATACAACATCCCCATGTCATCAG
Δ5/Δ5
AATGTGGGAGCCATACAGACATTCAGACAAATACAACATCCCCATGTCATCAG
b
Wild type
Δ5/+
Frequency KHz
Time (s)
dB
21-30
31-40
41-50
51-60
61-70
c
Vocalization times
+/+
Δ5/+
Δ5/Δ5
P5
P7
P9
P11
d
#30
Frequency KHz
Time (s)
dB
21-30
31-40
41-50
51-60
61-70

Figure S1

Gene name	*Chd5*	*Serpina1e*	*Stx18*	*Vmn2r110*	*Tcf7l2*
Chr. position	Chr4:152377125	Chr12:103951073	Chr5:38116152	Chr17:20574059	Chr19:55918961
Base change	G to T	A to C	G to T	T to C	T to C
Function	nonsynonymous	nonsynonymous	nonsynonymous	nonsynonymous	nonsynonymous
USVs at P7					
Few	○ (red)	○ (red)	○ (red)	○ (black)	○ (red)
Few	○ (red)	○ (red)	○ (red)		○ (red)
Few		○ (black)	○ (black)	○ (black)	○ (red)
Few					○ (red)
Few			○ (black)		○ (red)
Normal	○ (red)		○ (black)		○ (black)
Normal		○ (red)			○ (black)
Normal					○ (black)

Figure S2

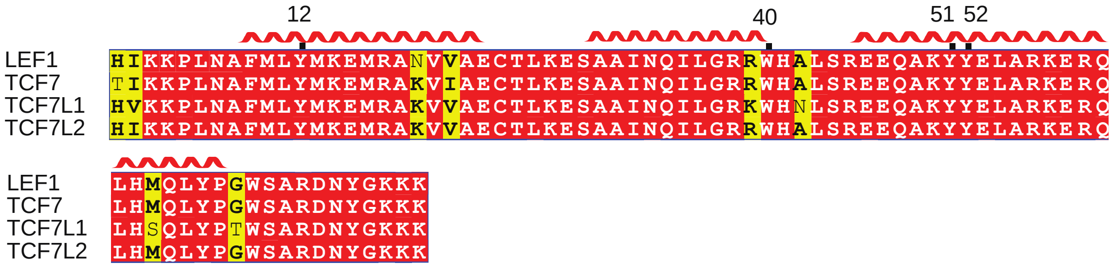
12
40
51 52
LEF1 HIKKPLNAFMLYMKEMRANVVAECTLKESAAINQILGRRWHALSREEQAKYYELARKERQ
TCF7 TIKKPLNAFMLYMKEMRAKVIAECTLKESAAINQILGRRWHALSREEQAKYYELARKERQ
TCF7L1 HVKKPLNAFMLYMKEMRAKVVAECTLKESAAINQILGRKWHNLSREEQAKYYELARKERQ
TCF7L2 HIKKPLNAFMLYMKEMRAKVVAECTLKESAAINQILGRRWHALSREEQAKYYELARKERQ
LEF1 LHMQLYPGWSARDNYGKKK
TCF7 LHMQLYPGWSARDNYGKKK
TCF7L1 LHSQLYPTWSARDNYGKKK
TCF7L2 LHMQLYPGWSARDNYGKKK

Figure S3

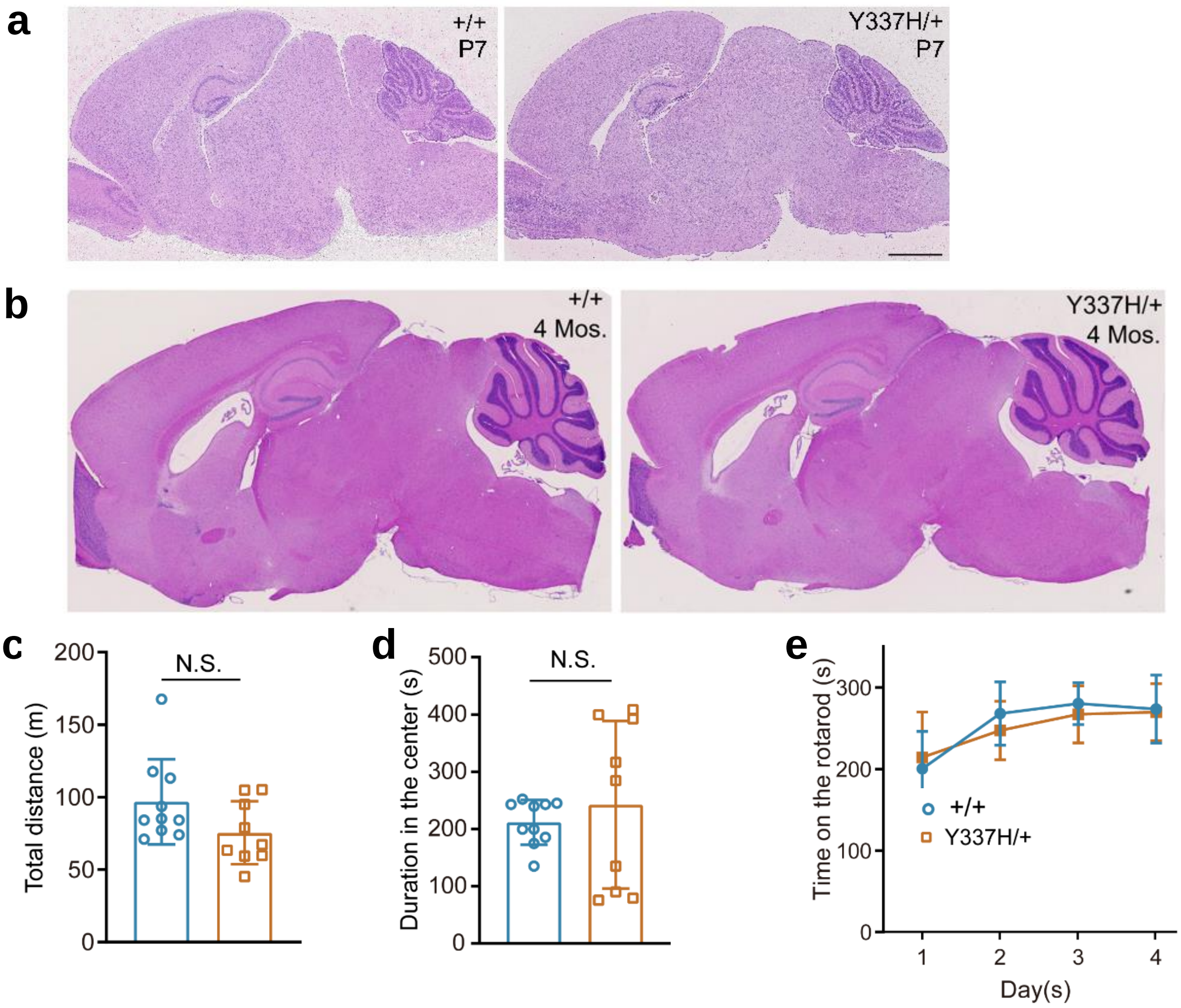
a
+/+
P7
Y337H/+
P7
b
+/+
4 Mos.
Y337H/+
4 Mos.
c
Total distance (m)
200
150
100
50
0
N.S.
d
Duration in the center (s)
500
400
300
200
100
0
N.S.
e
Time on the rotarod (s)
300
200
100
0
+/+
Y337H/+
1
2
3
4
Day(s)

Figure S4

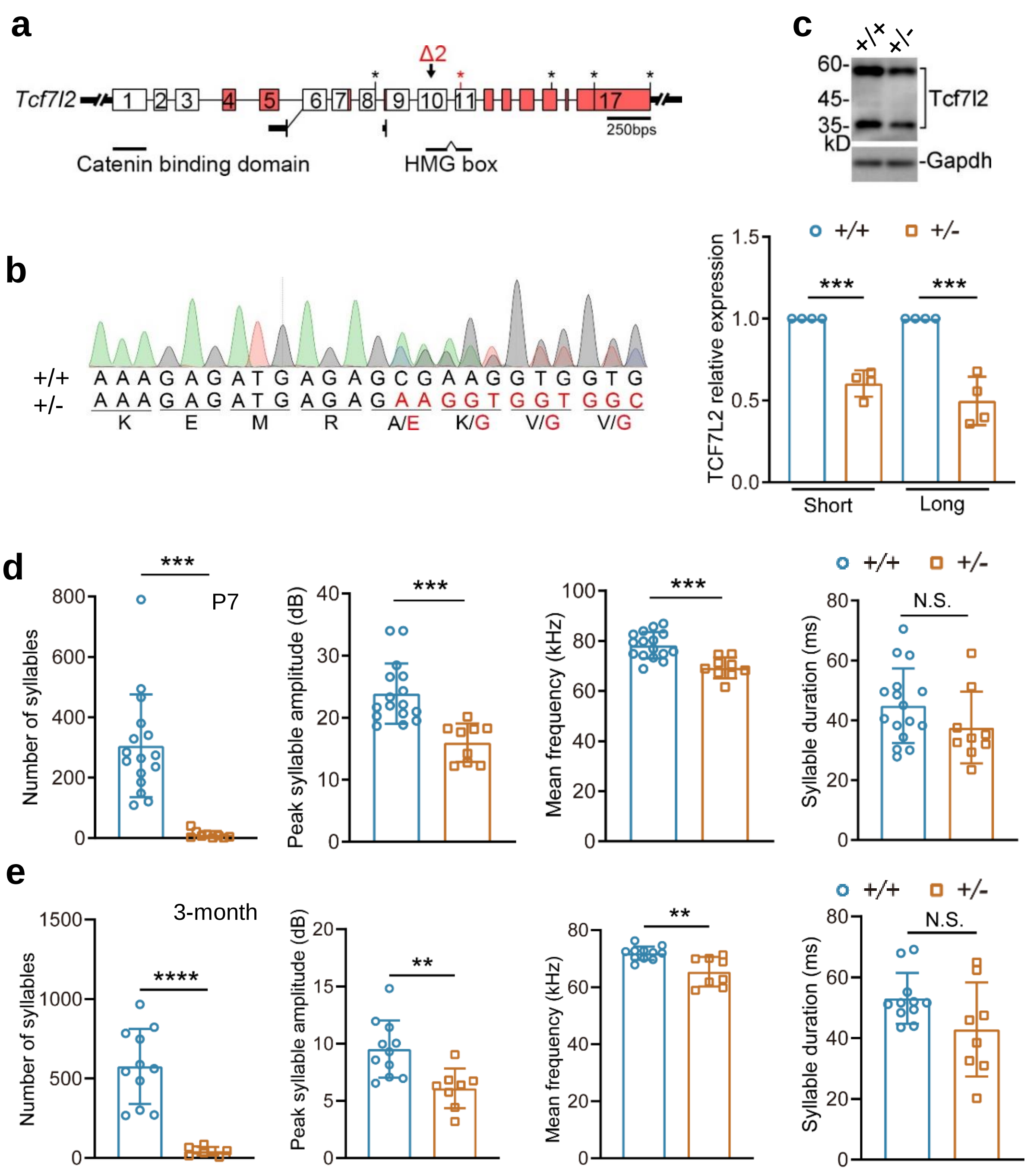
a
Tcf7l2
Δ2
1 2 3 4 5 6 7 8 9 10 11 17
250bps
Catenin binding domain
HMG box
b
+/+
+/-
A A A G A G A T G A G A G C G A A G G T G G T G
A A A G A G A T G A G A G A A G G T G G T G G C
K E M R A/E K/G V/G V/G
c
+/+ +/-
60-
45-
35-
kD
Tcf7l2
-Gapdh
+/+ +/-
TCF7L2 relative expression
1.5
1.0
0.5
0.0
***
***
Short
Long
d
***
P7
Number of syllables
800
600
400
200
0
Peak syllable amplitude (dB)
40
30
20
10
0
Mean frequency (kHz)
100
80
60
40
20
0
+/+ +/-
N.S.
Syllable duration (ms)
80
60
40
20
0
e
3-month
****
Number of syllables
1500
1000
500
0
**
Peak syllable amplitude (dB)
20
15
10
5
0
**
Mean frequency (kHz)
80
60
40
20
0
+/+ +/-
N.S.
Syllable duration (ms)
80
60
40
20
0

Figure S5

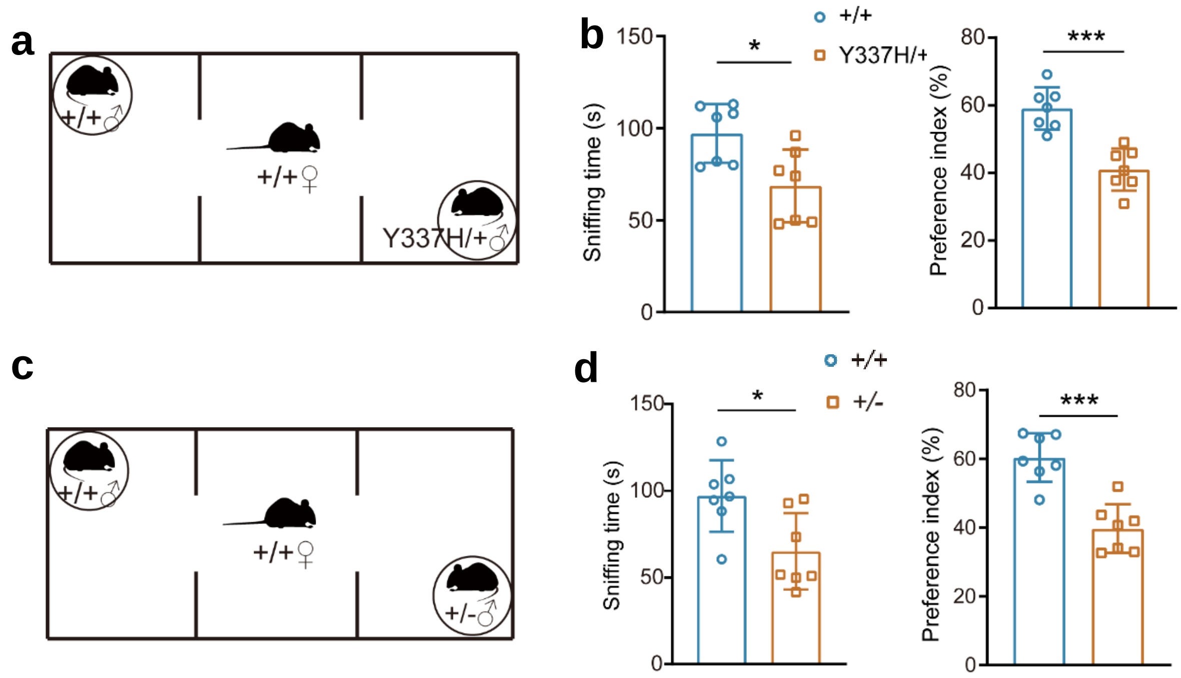
a
+/+♂
+/+♀
Y337H/+♂
b
150
100
50
0
Sniffing time (s)
*
+/+
Y337H/+
80
60
40
20
0
Preference index (%)
***
c
+/+♂
+/+♀
+/-♂
d
+/+
+/-
Sniffing time (s)
Preference index (%)

Figure S6

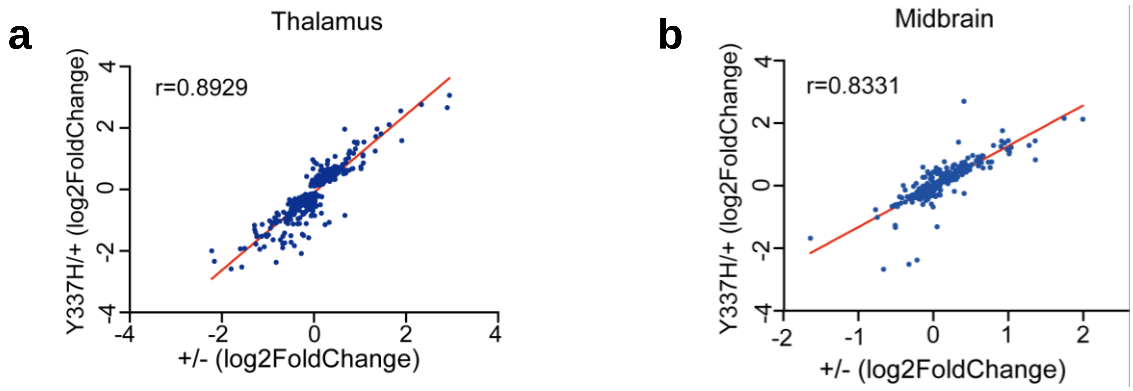
a
Thalamus
r=0.8929
Y337H/+ (log2FoldChange)
+/- (log2FoldChange)
4
2
0
-2
-4
-4
-2
0
2
4
b
Midbrain
r=0.8331
Y337H/+ (log2FoldChange)
+/- (log2FoldChange)
4
2
0
-2
-4
-2
-1
0
1
2

Figure S7

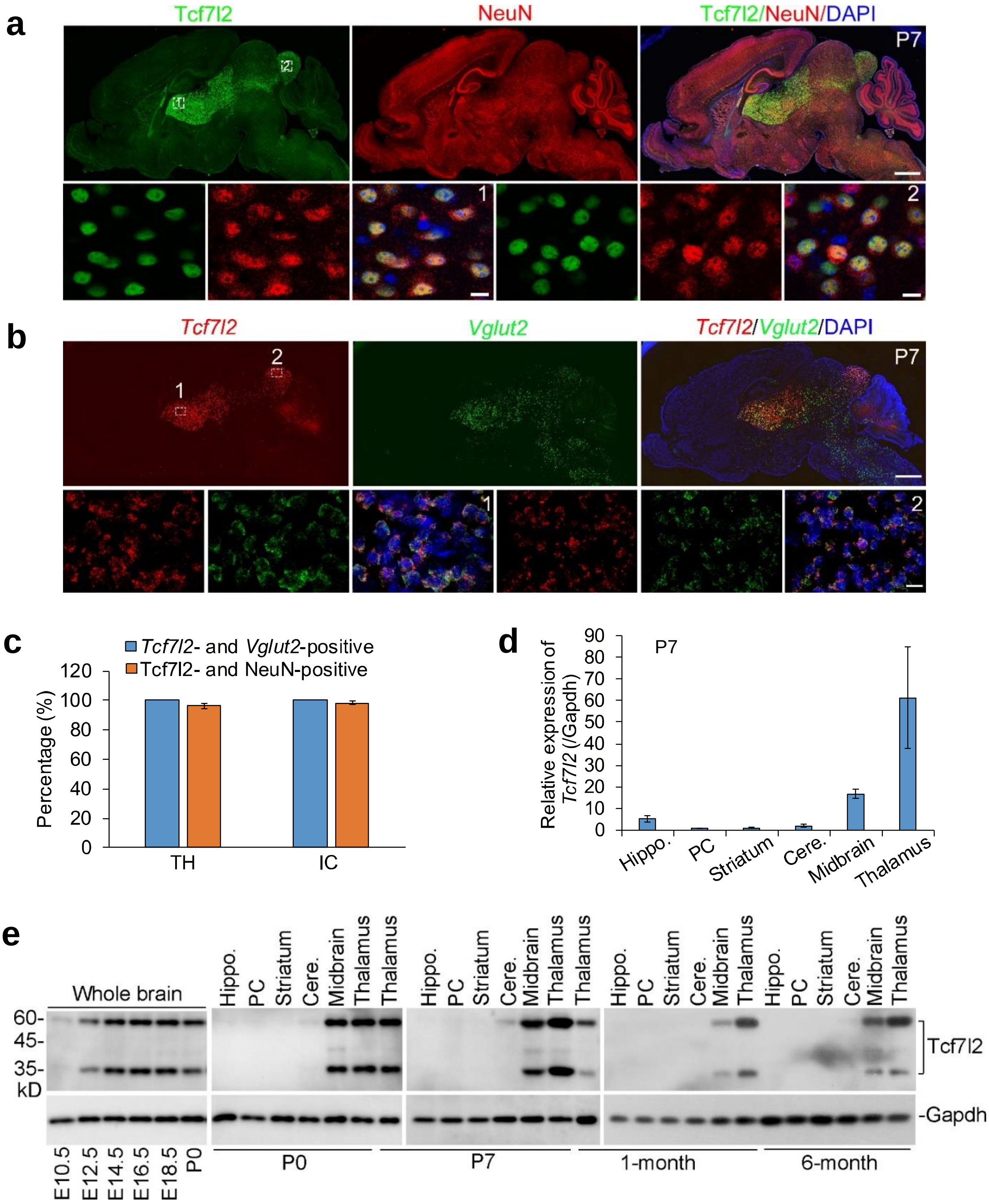
a
Tcf7l2
NeuN
Tcf7l2/NeuN/DAPI
P7
1
2
b
Tcf7l2
Vglut2
Tcf7l2/Vglut2/DAPI
P7
1
2
c
Tcf7l2- and Vglut2-positive
Tcf7l2- and NeuN-positive
Percentage (%)
120
100
80
60
40
20
0
TH
IC
d
P7
Relative expression of Tcf7l2 (/Gapdh)
90
80
70
60
50
40
30
20
10
0
Hippo.
PC
Striatum
Cere.
Midbrain
Thalamus
e
Whole brain
Hippo.
PC
Striatum
Cere.
Midbrain
Thalamus
60-
45-
35-
kD
Tcf7l2
-Gapdh
E10.5
E12.5
E14.5
E16.5
E18.5
P0
P0
P7
1-month
6-month

Figure S8

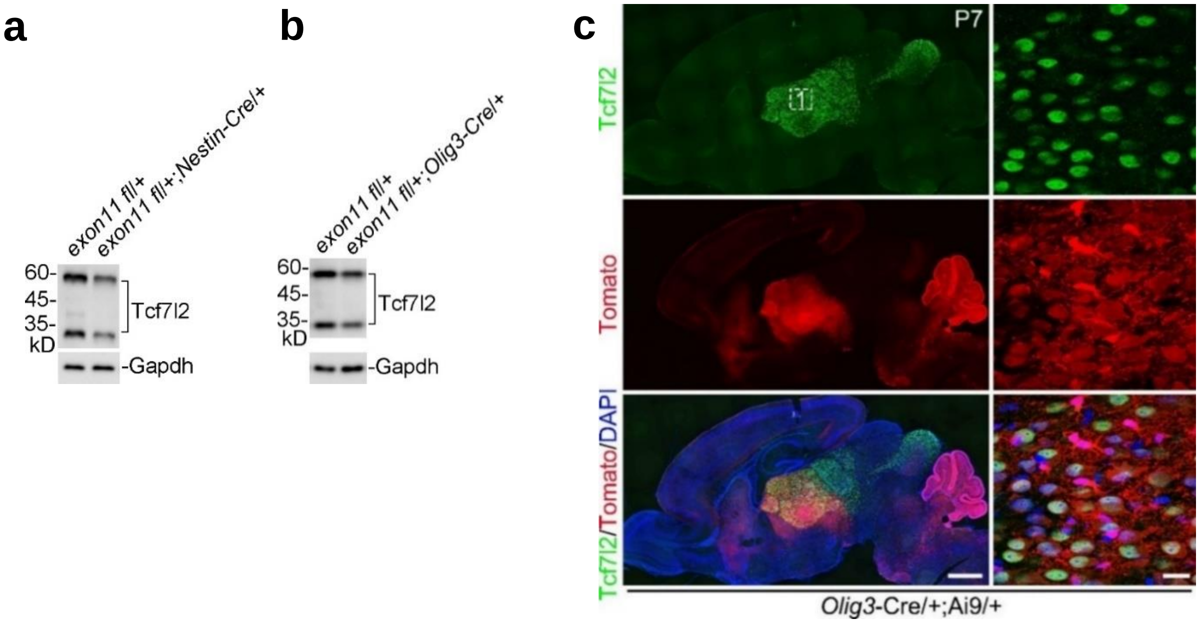
a
b
c
exon11 fl/+
exon11 fl/+;Nestin-Cre/+
60-
45-
35-
kD
Tcf7l2
-Gapdh
exon11 fl/+
exon11 fl/+;Olig3-Cre/+
60-
45-
35-
kD
Tcf7l2
-Gapdh
P7
Tcf7l2
Tomato
Tcf7l2/Tomato/DAPI
Olig3-Cre/+;Ai9/+

Figure S9

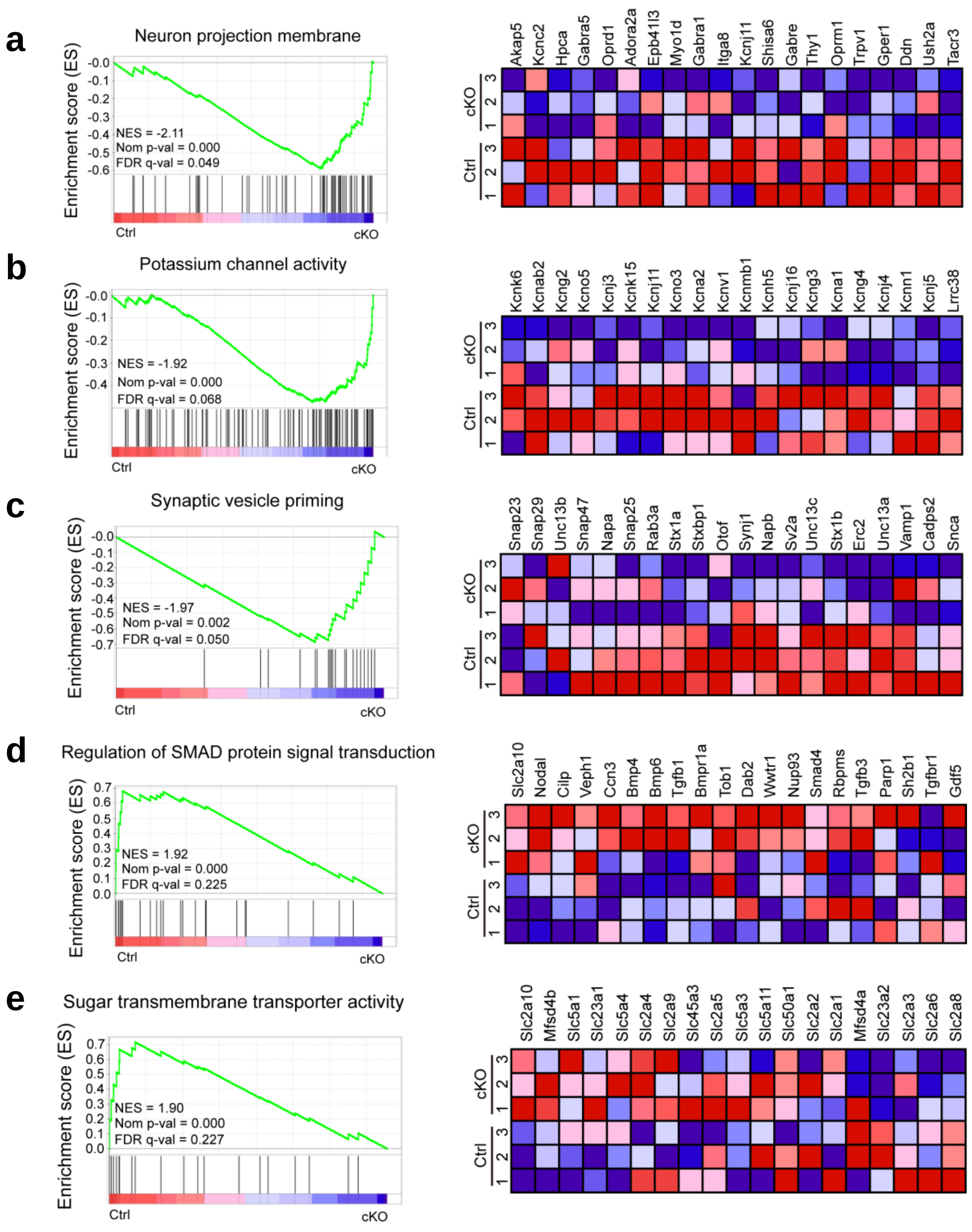
a
Neuron projection membrane
Enrichment score (ES)
NES = -2.11
Nom p-val = 0.000
FDR q-val = 0.049
Ctrl
cKO
Akap5 Kcnc2 Hpca Gabra5 Oprd1 Adora2a Epb41l3 Myo1d Gabra1 Itga8 Kcnj11 Shisa6 Gabre Thy1 Oprm1 Trpv1 Gper1 Ddn Ush2a Tacr3
b
Potassium channel activity
Enrichment score (ES)
NES = -1.92
Nom p-val = 0.000
FDR q-val = 0.068
Ctrl
cKO
Kcnk6 Kcnab2 Kcng2 Kcno5 Kcnj3 Kcnk15 Kcnj11 Kcno3 Kcna2 Kcnv1 Kcnmb1 Kcnh5 Kcnj16 Kcng3 Kcna1 Kcng4 Kcnj4 Kcnn1 Kcnj5 Lrrc38
c
Synaptic vesicle priming
Enrichment score (ES)
NES = -1.97
Nom p-val = 0.002
FDR q-val = 0.050
Ctrl
cKO
Snap23 Snap29 Unc13b Snap47 Napa Snap25 Rab3a Stx1a Stxbp1 Otof Synj1 Napb Sv2a Unc13c Stx1b Erc2 Unc13a Vamp1 Cadps2 Snca
d
Regulation of SMAD protein signal transduction
Enrichment score (ES)
NES = 1.92
Nom p-val = 0.000
FDR q-val = 0.225
Ctrl
cKO
Slc2a10 Nodal Cilp Veph1 Ccn3 Bmp4 Bmp6 Tgfb1 Bmpr1a Tob1 Dab2 Wwtr1 Nup93 Smad4 Rbpms Tgfb3 Parp1 Sh2b1 Tgfbr1 Gdf5
e
Sugar transmembrane transporter activity
Enrichment score (ES)
NES = 1.90
Nom p-val = 0.000
FDR q-val = 0.227
Ctrl
cKO
Slc2a10 Mfsd4b Slc5a1 Slc23a1 Slc5a4 Slc2a4 Slc2a9 Slc45a3 Slc2a5 Slc5a3 Slc5a11 Slc50a1 Slc2a2 Slc2a1 Mfsd4a Slc23a2 Slc2a3 Slc2a6 Slc2a8

Figure S10

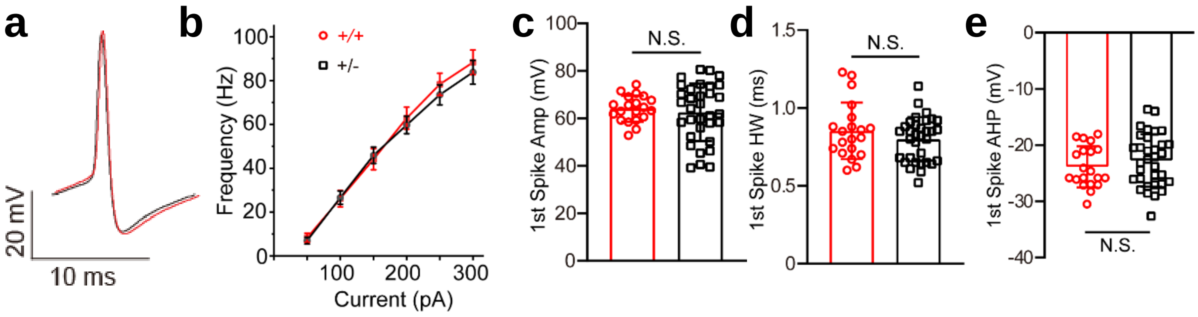
a
20 mV
10 ms
b
Frequency (Hz)
+/+
+/-
Current (pA)
c
1st Spike Amp (mV)
N.S.
d
1st Spike HW (ms)
N.S.
e
1st Spike AHP (mV)
N.S.

Figure S11

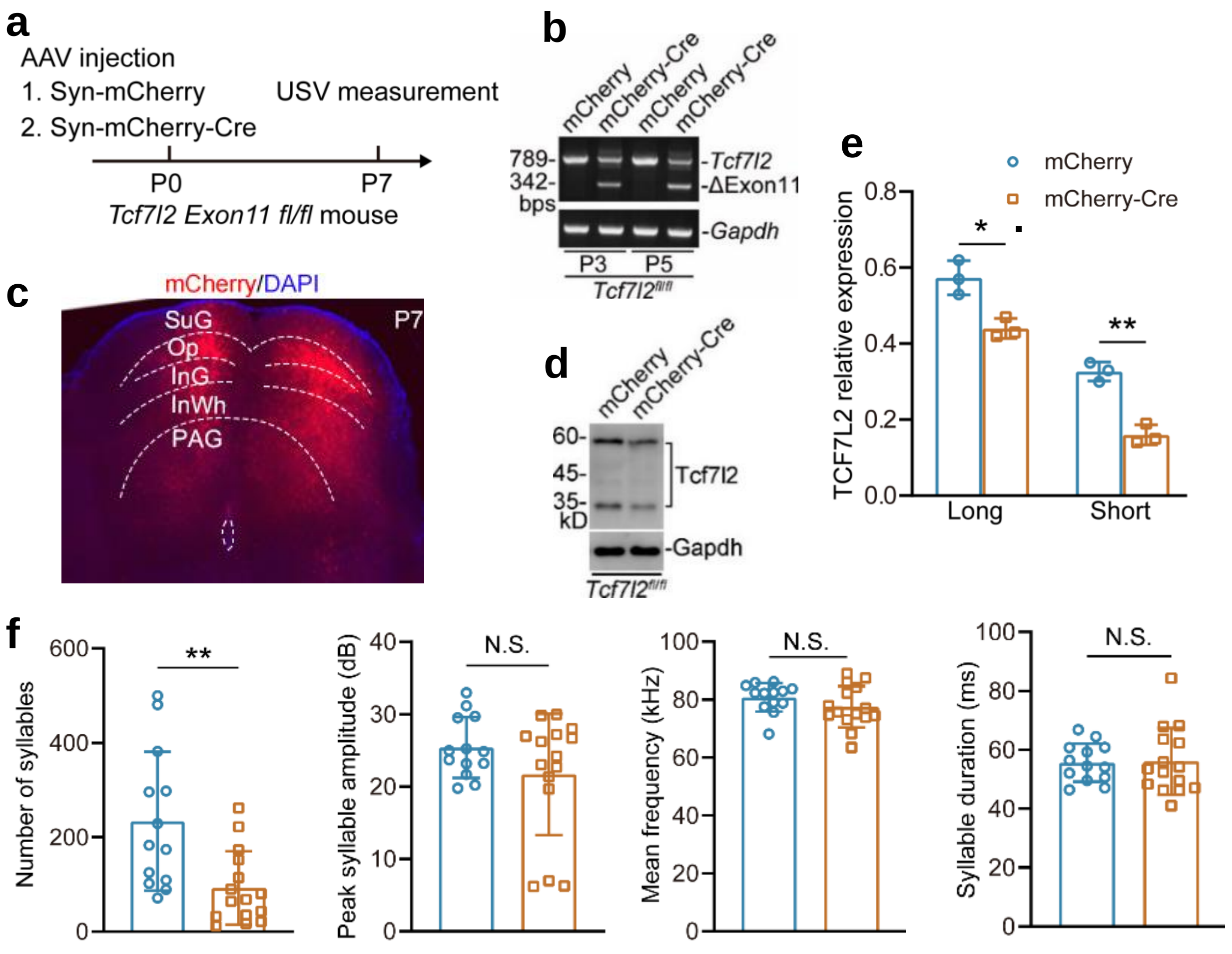
a
AAV injection
1. Syn-mCherry
2. Syn-mCherry-Cre
USV measurement
P0
P7
Tcf7l2 Exon11 fl/fl mouse
b
mCherry
mCherry-Cre
mCherry
mCherry-Cre
789-
342-
bps
-Tcf7l2
-ΔExon11
-Gapdh
P3
P5
Tcf7l2fl/fl
c
mCherry/DAPI
P7
SuG
Op
InG
InWh
PAG
d
mCherry
mCherry-Cre
60-
45-
35-
kD
Tcf7l2
-Gapdh
Tcf7l2fl/fl
e
mCherry
mCherry-Cre
TCF7L2 relative expression
0.0
0.2
0.4
0.6
0.8
*
**
Long
Short
f
Number of syllables
0
200
400
600
**
Peak syllable amplitude (dB)
0
10
20
30
40
N.S.
Mean frequency (kHz)
0
20
40
60
80
100
N.S.
Syllable duration (ms)
0
20
40
60
80
100
N.S.

Figure S12

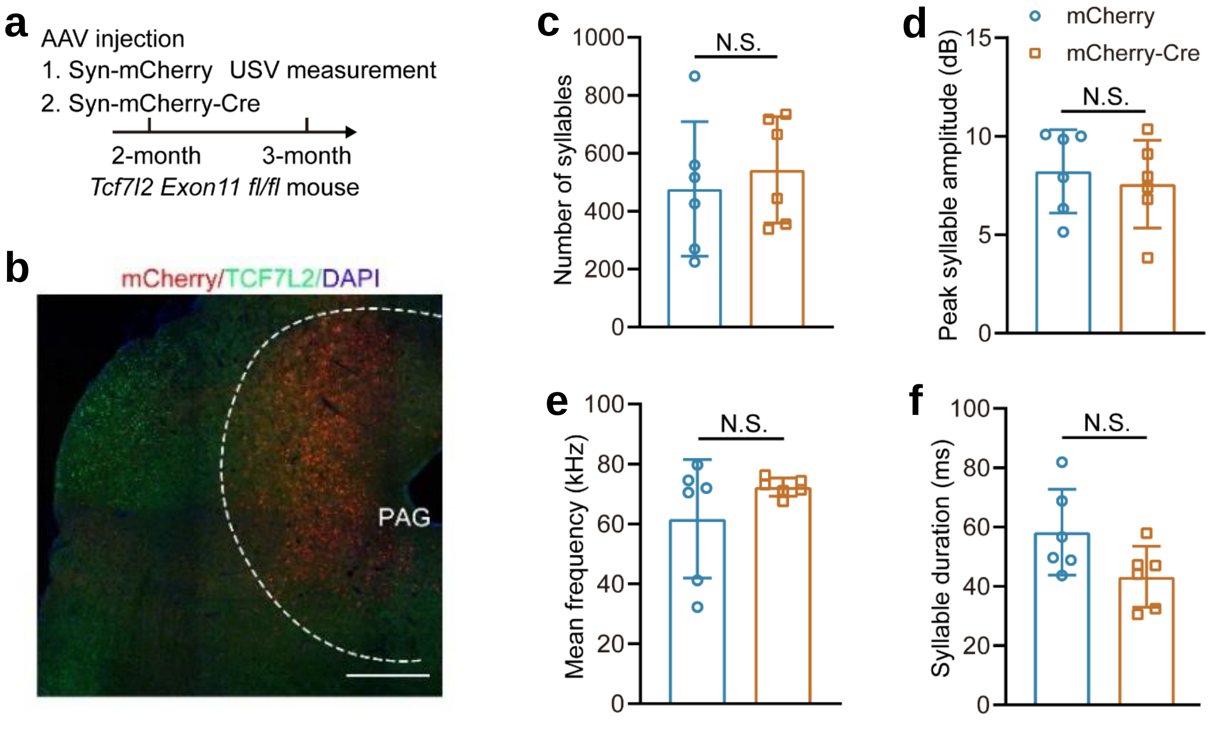
a
AAV injection
1. Syn-mCherry
2. Syn-mCherry-Cre
USV measurement
2-month
3-month
Tcf7l2 Exon11 fl/fl mouse
b
mCherry/TCF7L2/DAPI
PAG
c
Number of syllables
N.S.
d
Peak syllable amplitude (dB)
N.S.
mCherry
mCherry-Cre
e
Mean frequency (kHz)
N.S.
f
Syllable duration (ms)
N.S.

Figure S13

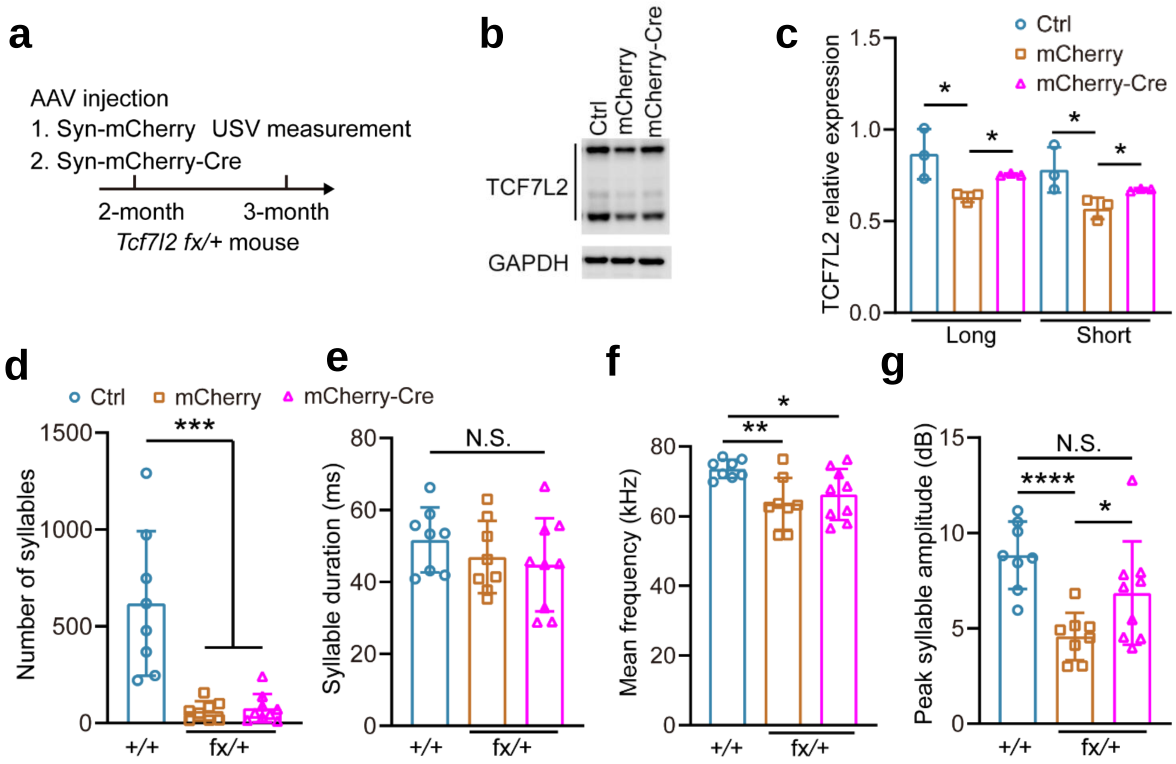
a
AAV injection
1. Syn-mCherry
2. Syn-mCherry-Cre
USV measurement
2-month
3-month
Tcf7l2 fx/+ mouse
b
Ctrl
mCherry
mCherry-Cre
TCF7L2
GAPDH
c
TCF7L2 relative expression
Ctrl
mCherry
mCherry-Cre
Long
Short
d
Number of syllables
***
+/+
fx/+
e
Syllable duration (ms)
N.S.
f
Mean frequency (kHz)
**
*
g
Peak syllable amplitude (dB)
N.S.
****
*

Figure S14

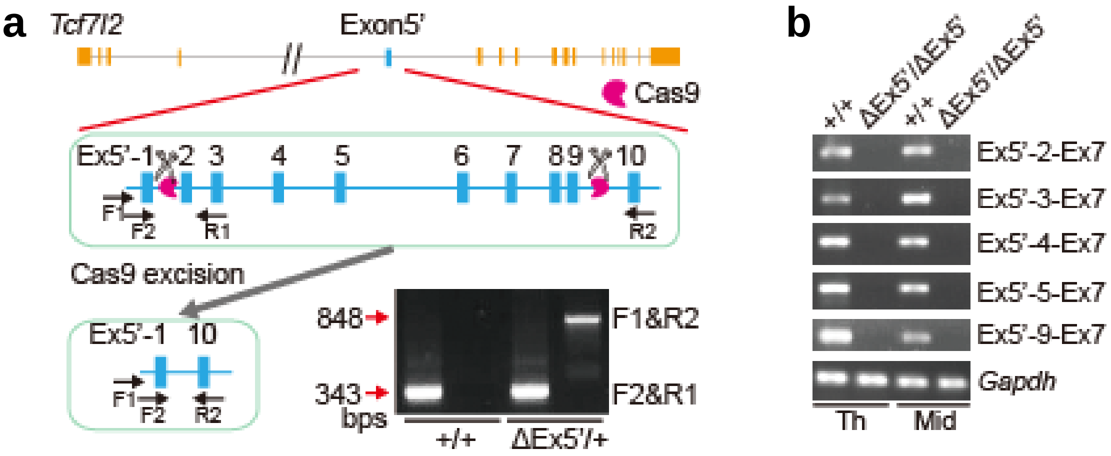
a
Tcf7l2
Exon5'
Cas9
Ex5'-1
2
3
4
5
6
7
8
9
10
F1
F2
R1
R2
Cas9 excision
Ex5'-1
10
F1
F2
R2
848
343
bps
F1&R2
F2&R1
+/+
ΔEx5'/+
b
+/+
ΔEx5'/ΔEx5'
+/+
ΔEx5'/ΔEx5'
Ex5'-2-Ex7
Ex5'-3-Ex7
Ex5'-4-Ex7
Ex5'-5-Ex7
Ex5'-9-Ex7
Gapdh
Th
Mid

Figure S15

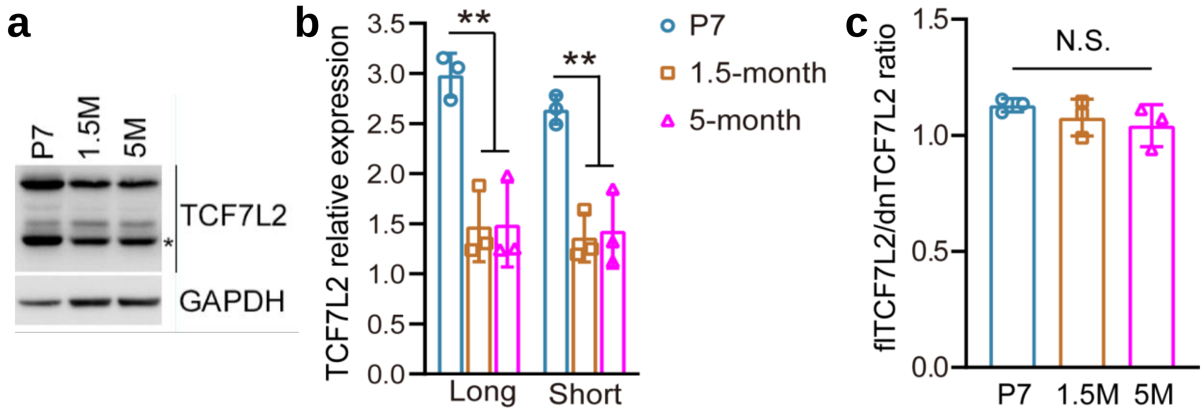
a
P7
1.5M
5M
TCF7L2
*
GAPDH
b
TCF7L2 relative expression
3.5
3.0
2.5
2.0
1.5
1.0
0.5
0.0
**
**
Long
Short
P7
1.5-month
5-month
c
flTCF7L2/dnTCF7L2 ratio
1.5
1.0
0.5
0.0
N.S.
P7
1.5M
5M

Figure S16

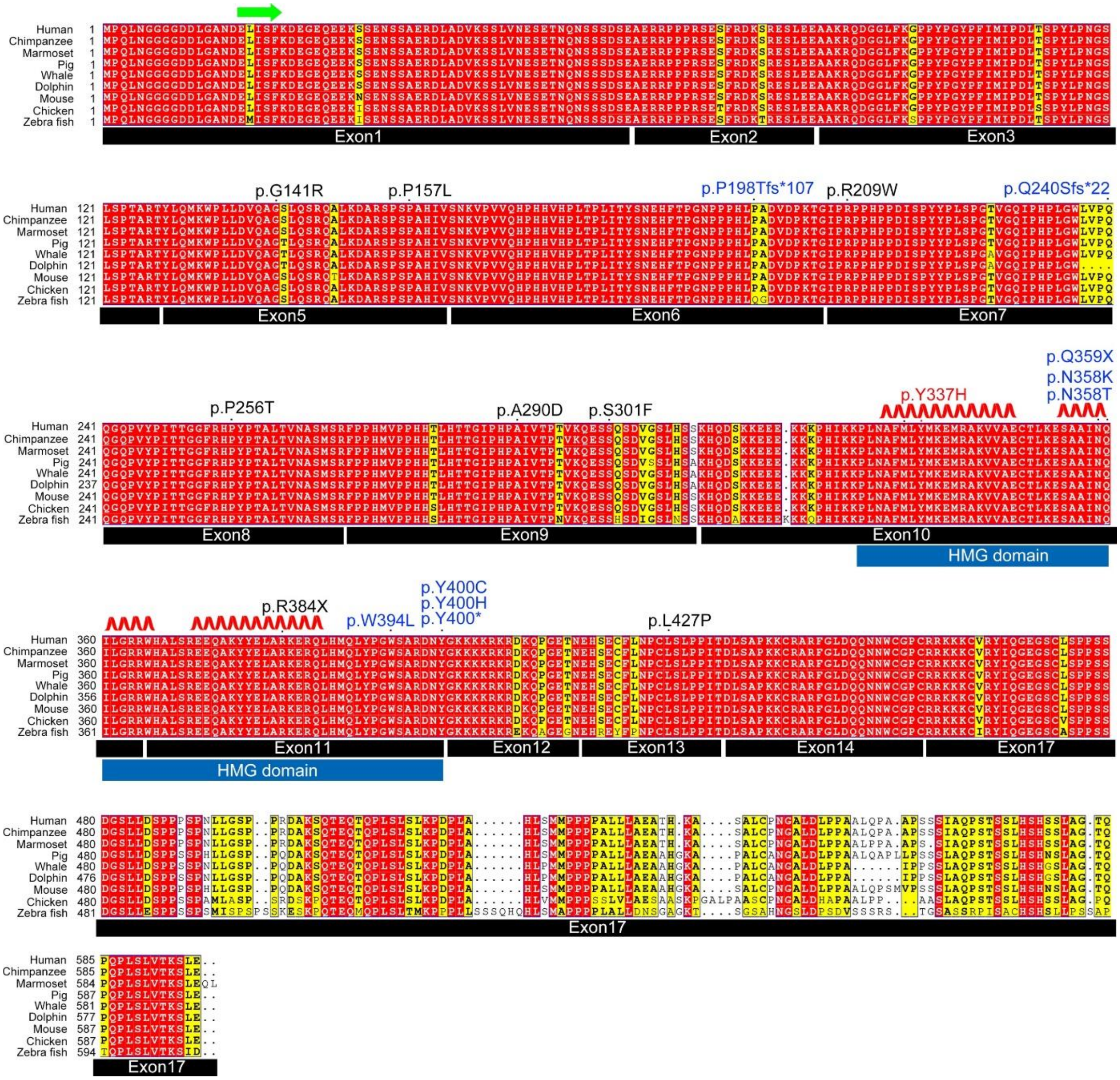
Human
Chimpanzee
Marmoset
Pig
Whale
Dolphin
Mouse
Chicken
Zebra fish
Exon1
Exon2
Exon3
p.G141R
p.P157L
p.P198Tfs*107
p.R209W
p.Q240Sfs*22
Exon5
Exon6
Exon7
p.Q359X
p.N358K
p.N358T
p.Y337H
p.P256T
p.A290D
p.S301F
Exon8
Exon9
Exon10
HMG domain
p.Y400C
p.Y400H
p.Y400*
p.R384X
p.W394L
p.L427P
Exon11
Exon12
Exon13
Exon14
Exon17
HMG domain
Exon17
Exon17

Figure S17

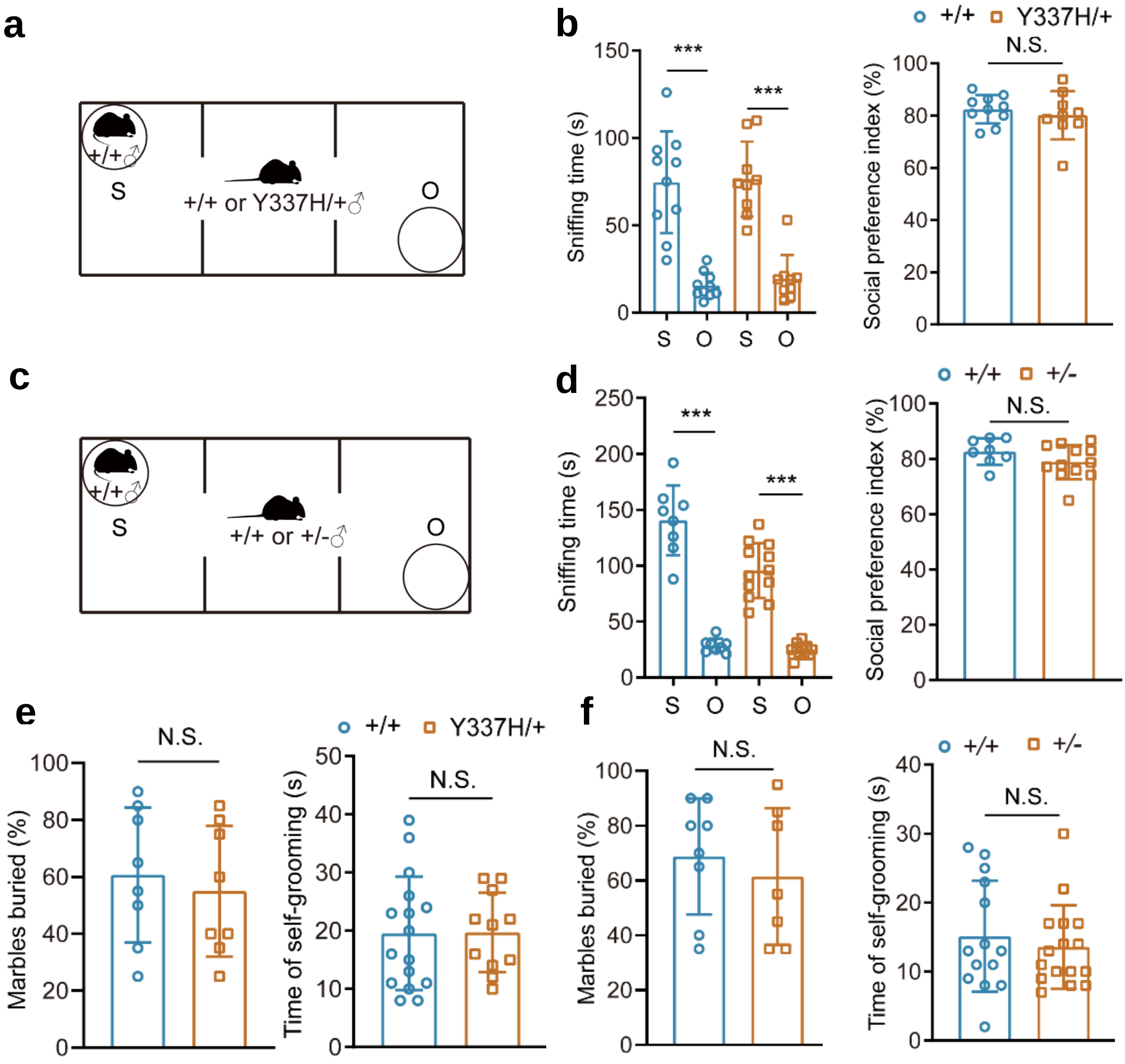
a
+/+♂
S
+/+ or Y337H/+♂
O
b
+/+
Y337H/+
***
***
Sniffing time (s)
150
100
50
0
S
O
S
O
N.S.
Social preference index (%)
100
80
60
40
20
0
c
+/+♂
S
+/+ or +/-♂
O
d
+/+
+/-
***
***
Sniffing time (s)
250
200
150
100
50
0
S
O
S
O
N.S.
Social preference index (%)
100
80
60
40
20
0
e
N.S.
Marbles buried (%)
100
80
60
40
20
0
+/+
Y337H/+
N.S.
Time of self-grooming (s)
50
40
30
20
10
0
f
N.S.
Marbles buried (%)
100
80
60
40
20
0
+/+
+/-
N.S.
Time of self-grooming (s)
40
30
20
10
0

Figure S18

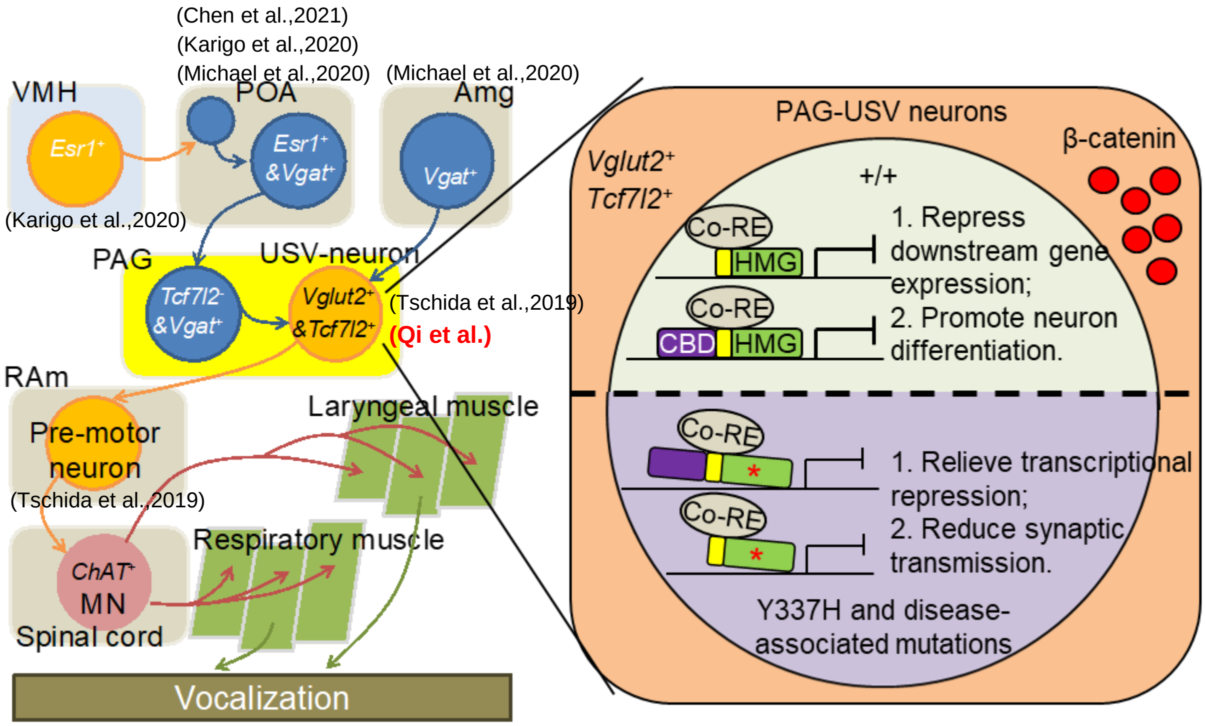
(Chen et al.,2021)
(Karigo et al.,2020)
(Michael et al.,2020)
(Michael et al.,2020)
VMH
POA
Amg
Esr1+
Esr1+ &Vgat+
Vgat+
(Karigo et al.,2020)
PAG
USV-neuron
Tcf7l2- &Vgat+
Vglut2+ &Tcf7l2+
(Tschida et al.,2019)
(Qi et al.)
RAm
Pre-motor neuron
(Tschida et al.,2019)
Laryngeal muscle
Respiratory muscle
ChAT+ MN
Spinal cord
Vocalization
PAG-USV neurons
Vglut2+
Tcf7l2+
β-catenin
+/+
Co-RE
HMG
CBD
1. Repress downstream gene expression;
2. Promote neuron differentiation.
1. Relieve transcriptional repression;
2. Reduce synaptic transmission.
Y337H and disease-associated mutations

Figure S19
