# Supplementary material for: TCF7L2 acts as a molecular switch in midbrain to control mammal vocalization through its DNA binding domain but not transcription activation domain
Source: Mol Psychiatry. 2023 Feb 13;28(4):1703–17. doi: 10.1038/s41380-023-01993-5 (PMC10208975; doi:10.1038/s41380-023-01993-5)
Supplement: Supplementary file 4 — Figure_Legends [file 41380_2023_1993_MOESM4_ESM.docx]

**Figure S1. *Foxp2* KO impairs mouse USV production.**

(a) A CRISPR/Cas9-based *Foxp2* KO mouse. The gRNA designed for the KO is located in *Foxp2* exon13 upstream of the exons coding Fox-head domain. A 5-nucleotide deletion (Δ5) generated by CRISPR/Cas9-mediated editing and the mouse homozygous for the deletion (Δ5/Δ5) was applied for the Sanger sequencing. The gRNA sequences were underlined and PAM (NGG) site was boxed.

(b) Pup USVs of the wildtype and heterozygous KO (Δ5/+) pups at P5 were measured by a USV detector (Med Associates Inc.). A representative USV impairment of Δ5/+ mouse P5 was shown.

(c) Both Δ5/+ and Δ5/Δ5 mice displayed the USV impairment at P5, 7, 9, and 11.

(d) Family members from #30 displayed USV impairment at P5.

In c, data are presented as mean ± SEM, +/+, n=15; Δ5/+, n=14; Δ5/Δ5, n=3, * *p*<0.05, ** *p*<0.01, t-test, SPSS.

**Figure S2. Identification of an ENU-induced *Tcf7l2* mutation that impairs pup USVs.**

Some of nonsynonymous mutations identified by whole-exome sequencing are shown, which were tested in 5 affected (Few USVs) and 3 unaffected (Normal USVs) mice in the #30 family. The *Tcf7l2* mutation was co-segregated with the USV impairment at P7. The red circle, mutations identified by sanger sequencing; black circle, mutations not identified.

**Figure S3. Aromatic residues Y12, W40, Y51, and Y52 in LEF1 HMG box are shared by the TCF/LEF family members.**

An alignment of HMG boxes of 4 human TCF/LEF family members and Y12, W40, Y51, and Y52 are highlighted.

**Figure S4. Normal brain morphologies and motor abilities shown in the ENU-induced mutant animals.**

(a and b) Hematoxylin-eosin staining of wildtype (+/+) and the ENU-induced mutant (Y337H/+) brains at P7 (a) and 4 months of age (b). Scale bar, 1mm.

(c-e) The mice with indicated genotypes were applied for Open field (c and d) and Rotarod tests (e). The value are presented as mean ± SD. In c and d, +/+, n=10; *Tcf7l2^Y337H/+^*, n=9; in e, +/+, n=12; *Tcf7l2^Y337H/+^*, n=12. Mice, male, 3 months of age. N.S., no significant difference, t-test, SPSS.

**Figure S5. Haploinsufficiency of *Tcf7l2* impairs pup USV production and adult mouse vocal communication.**

(a and b) A 2-nucleotide deletion (+/-) in *Tcf7l2* exon 10, which encodes part of TCF7L2 HMG domain, was introduced by Crispr/Cas9. The deletion leads to a premature termination codon (red asterisk) in *Tcf7l2.* DNA chromatogram (b) of the heterozygous KO mouse (+/-).

(c) The Δ2 deletion significantly reduces the expression of TCF7L2 in midbrain at P7. GAPDH as loading control. Short, low molecular weight (~35kD) TCF7L2; Long, high molecular weight (~60kD) TCF7L2.

(d and e) The key features of USVs produced by the wildtype (+/+) and heterozygous KO mutant (+/-) mice at P7 (d) and 3-month of age (e).

The value are presented as mean ± SD. In c, n=3; in d, +/+ (n=16), *Tcf7l2^+/-^* (n=9); in e, +/+ (n=11), *Tcf7l2^+/-^* (n=8). N.S., no significant difference, * *p*<0.05, *** *p*<0.001, t-test, SPSS.

**Figure S6. Both Y337H mutation and *Tcf7l2* heterozygous KO in male reduce female sexual preference.**

(a-d) Y337H and haploinsufficiency of *Tcf7l2* in male reduce female preference. Diagrams illustrates the female preference measurement by modification of three-chamber test (a and c). The data summary was shown in (b and d).

The value are presented as mean ± SD. In b, +/+ (male, n=7), WT (female, n=7), *Tcf7l2^Y337H/+^* (male, n=7); in d, +/+ (male, n=7), WT (female, n=7), *Tcf7l2^+/-^* (male, n=7). N.S., no significant difference, * *p*<0.05, *** *p*<0.001, t-test, SPSS.

**Figure S7. Similar RNA profiling of *Tcf7l2*^Y337H/+^ and *Tcf7l2*^+/-^ mice in brain.**

(a and b) RNA profiling of *Tcf7l2*^Y337H/+^ and *Tcf7l2*^+/-^ thalamus (a) and midbrain (b). Mouse, P7 (n = 3).

**Figure S8. Expression pattern of TCF7L2 protein and*Tcf7l2* mRNA in mouse brain.**

(a and b) Immunostaining of TCF7L2 and NeuN (a) and *in situ* hybridization of *Tcf7l2* and *Vglut2* (b) in a mouse sagittal section at P7. Enlarged region 1 (Thalamus, TH) and 2 (Inferior colliculus, IC) were shown (Lower).

(c) Statistical analysis of double positive neurons in region 1 (TH) and 2 (IC) at P7. (d) The expression of *Tcf7l2* in various brain tissues detected by the real-time PCR at P7.

(e) Expression pattern of TCF7L2 was examined by western blot. GAPDH, loading control.

In c and d, the value are presented as mean ± SEM (n=3). In a and b, scale bar, 1mm (low magnification) and 20 μm (high magnification).

**Figure S9. TCF7L2 expression pattern at P7.**

(a and b) The expression of TCF7L2 in the indicated cKO mice by western blot at P7. GAPDH, loading control.

(c) *Oligo3* expression was reported by Ai9 reporter mouse (PMID: 20023653). TCF7L2 expression was measured by immunostaining. Enlarged region 1 (Thalamus) was shown in the right. Scale bar, 1mm (left, low magnification) and 20 μm (right, high magnification).

**Figure S10. Significantly enriched pathways in PAG analyzed by GSEA between control and *Tcf7l2* cKO.**

(a-e) Significantly enriched biological pathways and their individual genes identified by GSEA analysis. Mouse, P7, *exon11 fl*/*fl* (control) or *exon11 fl*/*fl*;*Vglut2*-*Cre*/+ (cKO). In heatmap, blue and red, down- and upregulated genes in the cKO PAGs, respectively. NES, normalized enrichment score.

**Figure S11. Haploinsufficiency of *Tcf7l2* does not affect neuron intrinsic excitability in LPAG.**

(a) Representative traces of action potentials (AP) in *+/+* and *Tcf7l2*^+/-^ mouse.

(b) AP frequency of LPAG neurons in *+/+* and *Tcf7l2*^+/-^ mouse in response to increasing depolarizing current.

(c-e) AP amplitude (c), AP half-width (d), and afterhyperpolarization (e) of the first AP evoked by current injection are comparable between +/+ and *Tcf7l2*^+/-^ mice. In b-e, the value are presented as mean ± SD (+/+, n=20/4; +/-, n=33/3). N.S., no significant difference, t-test, SPSS. HW, half-width; AHP, afterhyperpolarization.

**Figure S12. Expression of TCF7L2 in PAG is required for pup USV production.**

(a) A schematic diagram for *Tcf7l2* KO in PAG by AAV-Syn-mCherry-Cre injection. The AAV viral particles were injected into PAGs of *Tcf7l2* *exon11* *fl*/*fl* mice at P0 and USVs were measured at P7. The Cre expression was driven by a neuronal Syn1 promoter. AAV-Syn-mCherry served as a control.

(b) The removal of exon 11 (ΔExon11) at DNA level was detected in midbrain at P3 and P5 after AAV-mCherry-Cre injection. Gapdh served as a control for genomic DNA PCR.

(c) The mCherry expression indicated the AAV infected region at P7. We speculate that the infected regions above PAG are caused by PAG neural progenitors proliferation and migration.

(d and e) Expression of TCF7L2 in PAG of *Tcf7l2* *exon11 fl/fl* mice at P7 after AAV-mCherry or AAV-mCherry-Cre injections. Data summary was shown in (j) (n=3).

(f) Pup USV features were measured in *Tcf7l2 exon11 fl/fl* mice injected with AAV-mCherry and AAV-mCherry-Cre.

The value are presented as mean ± SD. N.S., no significant difference, ** *p*<0.01, *** *p*<0.001, t-test or ANOVA, SPSS. In f, AAV-mCherry (n=13), AAV-mCherry-Cre (n=15).

**Figure S13. TCF7L2 adulthood expression in PAG is not required for adult mouse vocal communication.**

(a) A schematic diagram for *Tcf7l2* KO in PAG by AAV-Syn-mCherry-Cre injection. The AAV viral particles were injected into PAG of *Tcf7l2* *exon11* *fl*/*fl* mouse at 2-month and USVs were measured at 3-month of age. The Cre expression was driven by a neuronal Syn1 promoter. AAV-Syn-mCherry served as a control.

(b) Immunostaining results displayed *Tcf7l2* KO in PAG by the AAV injection.

(c-f) Key features of USVs measured in the *Tcf7l2* conditional KO mice at 3-month of age.

The value are presented as mean ± SD. N.S., no significant difference, ** *p*<0.01, *** *p*<0.001, t-test or ANOVA, SPSS. In c-f, AAV-mCherry (n=6), AAV-mCherry-Cre (n=6).

**Figure S14. TCF7L2 adulthood expression in PAG is not sufficient for adult mouse vocal communication.**

(a) A schematic diagram for AAV-Syn-mCherry-Cre injection in PAG in *fx/+* mice. The AAV viral particles were injected into PAG of *Tcf7l2* *fx/+* mouse at 2-month and USVs were measured at 3-month of age. The Cre expression was driven by a neuronal Syn1 promoter. AAV-Syn-mCherry served as a control.

(b and c) The expression of TCF7L2 in the indicated AAV-injected mice (3-month of age) by western blot. GAPDH, loading control.

(d-g) Key features of USVs measured in *Tcf7l2 fx/+* mice after injections of AAV-mCherry and AAV-mCherry-Cre.

The value are presented as mean ± SD. N.S., no significant difference, ** *p*<0.01, *** *p*<0.001, t-test or ANOVA, SPSS. In b and c, AAV-mCherry (n=3), AAV-mCherry-Cre (n=3), in d-g, control (+/+, n=8), AAV-mCherry (n=8), AAV-mCherry-Cre (n=9).

**Figure S15. Generation of *Tcf7l2* exon5’ KO mouse.**

(a) A ~6kb DNA fragment containing exon5’-2 to -9 was removed by CRISPR/Cas9-based KO. We employed two sgRNAs (labeled as scissors). Ten alternative exon5’ of *Tcf7l2* are represented as blue rectangles. Primers for genotyping are labeled (F1, R1, F2, and R2). An additional 848bp band was only amplified by genomic DNA PCR in mouse heterozygous for exon5’ KO (ΔEx5’/+) but not +/+.

(b) Expression of exon5’-containing transcripts detected in +/+ but not ΔEx5’/ΔEx5’ mouse.

**Figure S16. The ratio of flTCF7L2 to dnTCF7L2 during midbrain development.**

(a and b) Both flTCF7L2 and dnTCF7L2 at 1.5-month and 5-month of age in midbrain were significantly lower that of P7. GAPDH, loading control. *, dnTCF7L2.

(c) The ratio of flTCF7L2 to dnTCF7L2 at P7, 1.5-month, and 5-month of age shows no significant change during midbrain development.

The value are presented as mean ± SD. Mouse, n=3. N.S., no significant difference, ** p<0.01, t-test, SPSS.

**Figure S17. TCF7L2 disease-associated mutations.**

Protein sequence alignment of TCF7L2 across various species and human TCF7L2 (NM_001198528) were employed for positioning. Disease-associated mutations include: 1) ENU-induced Y337H mutation colored in red; 2) mutations described in previous studies are colored in black (PMID: 25363768, 25533962, and 28191889) and blue (PMID:34003604), respectively.

**Figure S18. *Tcf7l2*^Y337H/+^ mutant and *Tcf7l2*^+/-^ mice have normal performance in three-chamber and stereotyped behavior tests.**

(a-d) Three-chamber test for animals with indicated genotypes. Diagrams for the tests (a and c). Social preference index was calculated as previously described (PMID: 31780330). S, stranger; O, object.

(e and f) Marble burying test and self-grooming measurement with indicated genotypes.

The value are presented as mean ± SD. N.S., no significant difference, *** *p*<0.001, t-test, SPSS. In b and d, +/+ (n=8-10), *Tcf7l2*^+/-^ (n= 12), and *Tcf7l2*^Y337H/+^ (n= 9). In e, for marble burying test, +/+ (n=8) and *Tcf7l2*^Y337H/+^ (n= 8); for self-grooming test, +/+ (n=16) and *Tcf7l2*^Y337H/+^ (n= 11). In f, for marble burying test, +/+ (n=8) and *Tcf7l2*^+/-^ (n= 7); for self-grooming test, +/+ (n=16) and *Tcf7l2*^+/-^ (n= 14).

**Figure S19. Our working model.**

Recently, lateral PAG-USV neurons (*Vglut2*+, excitatory) have been identified and characterized, which are necessary and sufficient for mouse USV production (PMID31204083). Besides PAG-USV neurons, *Esr1*-positive neurons in VMH (*Esr1*+) and POA (*Esr1*+ & *Vgat*+) and *Vgat*-positive neurons in Amg (*Vgat*+) and PAG (*Vgat*+) act upstream of PAG-USV neurons to contribute to mouse USV production and persistence (PMID: 33790464, 30900143, 33268894, and 33372655). PAG gates the vocal patterning networks located in caudal brainstem, including RAm, which project to vocal motor neuron (MN, *ChAT*+) pool in spinal cord to control laryngeal and respiratory muscles. The PAG to the brain stem hardware is crucial for both innate and learned vocalization. Here, we demonstrate that *Tcf7l2* is expressed in *Vglut2*+ but not *Vgat*+ (inhibitory) neurons in lateral PAG. Expression of *Tcf7l2* in *Vglut2*+ but not *Esr1*+ or *ChAT*+ neurons is essential for mouse vocalization. Removal of *Tcf7l2* in *Vglut2*+ neurons in midbrain impairs the USV production and lateral PAG synaptic transmission (Left). In PAG-USV neurons, both flTCF7L2 (containing CBD and HMG domains) and dnTCF7L2 (only containing HMG domain) are expressed and required for mouse USV production, suggesting that a transcriptional repression mechanism of TCF7L2 is crucial for mammal vocalization. We speculate that the transcriptional repression promotes neuronal differentiation. However, loss-of-function mutation Y337H and other disease-associated non-synonymous mutations in HMG domain (* in red) relieve the transcriptional repression and reduce neuronal differentiation in these neurons, which in turn decrease synaptic transmission in PAG-USV neurons and leads to impairments of vocal production and syllable complexity (Right). Abbreviations: PAG, periaqueductal gray; VMH, ventromedial hypothalamus; POA, preoptic area; Amg, amygdala; RAm, nucleus retroambiguus; Co-RE, co-repressors.
